# The Use of Clear Aligner Therapy for Orthognathic Surgery Patients: A Cross-Sectional Survey Among Saudi Orthodontists and Oral and Maxillofacial Surgeons

**DOI:** 10.12688/f1000research.170095.3

**Published:** 2026-04-15

**Authors:** Ahmad Salem Assari, Muslat A Bin Rubaia’an, Diaa Taisir Al-Marhoun, Hayat Arafah, AlAnood N Bin Saedan, Hajar Dar Salamah, Raneem Mohammed Aljomiey

**Affiliations:** 1 Department of Dentistry, King Faisal Specialist Hospital and Research Centre, Riyadh, Saudi Arabia; 2 Department of Oral and Maxillofacial Surgery and Diagnostic Sciences, College of Medicine and Dentistry, Riyadh Elm University, Riyadh, Riyadh Province, Saudi Arabia; 3 Department of Maxillofacial Surgery, Prince Sultan Military Medical City, Riyadh, Riyadh Province, Saudi Arabia; 4 Dental Clinic, Riyadh, Saudi Arabia; 5 Alwattan medical complex 3, Riyadh, Saudi Arabia; 6 Saudi Board in Periodontics, College of Medicine and Dentistry, Riyadh Elm University, Riyadh, Riyadh Province, Saudi Arabia

**Keywords:** Orthognathic surgery, Clear aligner therapy (CAT), Orthodontics, Oral and maxillofacial surgery, Saudi Arabia, Surgical orthodontics

## Abstract

**Background:**

Orthognathic surgery corrects severe skeletal discrepancies that cannot be addressed by orthodontics alone. With advancements in digital workflows and CAD/CAM technologies, clear aligner therapy (CAT) has emerged as a potential adjunct in surgical cases. However, its clinical application remains under-researched. This study aimed to exploratorily assess the knowledge, clinical experience, and attitudes of orthodontists and oral and maxillofacial surgeons in Saudi Arabia regarding CAT in orthognathic surgery patients.

**Methods:**

A cross-sectional survey was conducted from April 1 to May 31, 2023, using a closed-ended online questionnaire distributed to specialists across Saudi Arabia. The survey gathered demographic and clinical data. Statistical analysis included descriptive methods, chi-square testing, and univariate regression. Given the limited sample size, inferential analyses were performed for exploratory purposes and interpreted cautiously.

**Results:**

Out of 278 invited participants, 46 complete responses (16.5%) were received, which was substantially lower than the calculated minimum sample size. Most participants were aged 31–40 years and had 6–10 years of experience. Orthodontists accounted for 65.2% of the respondents, with oral and maxillofacial surgeons at 34.8%. While most treated 1–4 orthognathic patients monthly, 80% had no experience using CAT in these cases Although CAT was commonly used in routine orthodontic practice, its application in orthognathic surgery cases was limited. Exploratory regression analysis suggested a possible association between clinician age and CAT usage (R ^2^ = 0.38), whereas gender and years of experience did not show clear associations.

**Conclusion:**

This exploratory survey suggests that clinical experience with CAT in orthognathic surgery remains limited among surveyed clinicians in Saudi Arabia. The findings highlight perceived challenges related to surgical workflows, including pre-surgical alignment, intraoperative fixation, and postoperative occlusal control. Given that the achieved sample size (n = 46) was substantially lower than the calculated minimum requirement (n = 302), the results should be interpreted as preliminary observations and exploratory findings rather than definitive conclusions.

## Introduction

Orthognathic surgery remains the treatment of choice for adults with severe dentofacial deformities or skeletal discrepancies that cannot be corrected by orthodontic camouflage alone. ^1, 2^ Successful outcomes require a coordinated interdisciplinary approach involving both orthodontists and oral and maxillofacial surgeons. ^1, 2^

Orthodontic management for surgical cases traditionally involves pre-surgical decompensation using fixed appliances followed by post-surgical refinement. This study primarily refers to the conventional orthodontics-first approach, where pre-surgical orthodontic preparation is essential prior to orthognathic surgery. Although surgery-first protocols have been introduced in recent years, they eliminate or reduce the need for extensive pre-surgical orthodontics and therefore involve different clinical considerations. As such, the present investigation focuses on clinician perspectives within the orthodontics-first framework, where the role of clear aligner therapy in pre- and post-surgical phases remains clinically relevant. Fixed appliances, typically metal or ceramic brackets connected by archwires, remain the gold standard in this context due to their ability to deliver controlled tooth movements in all three planes of space. ^3, 4^ However, growing aesthetic demands, changing patient preferences, and concerns about oral hygiene and periodontal health have contributed to decreased patient acceptance of fixed appliances. ^5– 7^

The introduction of clear aligner therapy (CAT) in the late 20th century has provided an alternative, especially for patients prioritizing aesthetics and comfort. ^8^ Initially indicated for mild to moderate malocclusions, CAT has evolved considerably with advances in digital imaging, 3D printing, and biomechanical modeling, allowing treatment of increasingly complex dental movements. ^1, 6, 9– 11^ CAT now features transparent, removable trays generated through digital treatment planning, offering improved aesthetics, patient comfort, oral hygiene, and reduced chair time. ^7, 8^ Its use in combination with surgery is emerging; however, its role differs between orthodontics-first and surgery-first protocols. In orthodontics-first approaches, CAT may be used in pre- and post-surgical phases, whereas in surgery-first protocols its role is more limited and evolving. ^7^

Meanwhile, in Saudi Arabia, the demand for clear aligners has risen in tandem with global trends as patients increasingly seek orthodontic solutions that are less conspicuous and more comfortable than traditional braces. ^12^ However, there is little evidence exploring clinicians’ readiness, experience, or confidence in integrating CAT into surgical orthodontics. This gap in empirical data limits understanding of how practitioners perceive its feasibility in complex surgical workflows. ^13^^,^^14^ This knowledge gap presents a barrier for both orthodontists and oral and maxillofacial surgeons in utilizing CAT effectively for complex skeletal discrepancies. Region-specific studies indicate that orthognathic surgery is among the most frequently performed oral and maxillofacial procedures in Saudi teaching hospitals, ^15^ and patient motivation is often driven by aesthetic concerns, with satisfaction rates exceeding 90%. ^16, 17^ Moreover, research highlights anatomical variations between regions within the country such as greater microgenia and longer facial profiles in southern populations which further emphasizes the need for personalized surgical planning and broader adoption of advanced, adaptable technologies like CAT. ^13, 18^

Given the rising popularity of CAT and the evolving landscape of orthodontic practice, there is a need to evaluate clinician perspectives in surgical contexts. The current study aimed to assess the knowledge, clinical experience, attitudes, and practices of Saudi orthodontists and oral and maxillofacial surgeons regarding the use of clear aligner therapy in orthognathic surgery patients.

## Methodology

### Study design, setting and ethical consideration

This cross-sectional study was ethically approved by the Institutional Review Board (IRB) of Riyadh Elm University (Approval No. [FUGRP/2023/303/929]) and conducted in full accordance with the Declaration of Helsinki. All participants received detailed information about the study objectives, procedures, and data confidentiality, and provided electronic informed consent prior to participation. Participation was voluntary, and anonymity and data confidentiality were maintained throughout.

A closed-ended, self-administered questionnaire was designed online using Google Forms and distributed among orthodontists and maxillofacial surgeons practicing in Saudi Arabia. It was distributed among orthodontists and maxillofacial surgeons practicing in Saudi Arabia. The survey was conducted between April 1 and May 31, 2023. Participants were recruited through professional email lists, social media platforms, and direct distribution via professional organizations, including the Saudi Orthodontic Society and regional oral and maxillofacial surgery networks. To minimize sampling bias, invitations were sent broadly to licensed practitioners across academic, private, and governmental sectors.

### Sample size and population

According to recent data from the Saudi Commission for Health Specialties and regional workforce reports, there are an estimated 900–1,100 orthodontists and 400–500 oral and maxillofacial surgeons actively practicing in Saudi Arabia. ^19^ Our sampling frame of 278 clinicians was drawn from professional networks and academic institutions, covering a cross-section of these specialties. The required sample size was calculated using the standard formula for a finite population:

$$n=\frac{Z^{2}\times p\times q\times N}{e^{2}\left(N-1\right)+Z^{2}\times p\times q}$$

Where:

•
n = required sample size
•
N = total population of orthodontists and oral and maxillofacial surgeons in Saudi Arabia (≈ 1,400)
•
Z = 1.96 (for 95% confidence level)
•
p = 0.5 (assumed proportion)
•
q=1−p

•
e = 0.05 (margin of error)

Based on these parameters, the minimum required sample size was *n* = 302. Although the theoretical minimum sample size was calculated as 302 participants, the final number of complete responses obtained was lower due to the voluntary nature of survey participation and the specialized target population. Consequently, the present study should be interpreted as an exploratory survey investigation rather than a fully powered inferential study. The statistical analyses were therefore conducted primarily to identify preliminary patterns and associations rather than to support definitive population-level conclusions. To maximize recruitment and account for expected non-response, the questionnaire was distributed to 278 clinicians who were accessible through professional networks, academic contacts, and professional organizations such as the Saudi Orthodontic Society and regional oral and maxillofacial surgery networks.

A non-probability convenience sampling method was used, which is appropriate for exploratory research involving geographically dispersed professional populations. Given the niche focus on surgical orthodontics and CAT, targeted sampling via professional channels was necessary to reach experienced clinicians. While convenience sampling may introduce selection bias, it remains an accepted approach in preliminary, perception-based clinical surveys. This approach may introduce **self-selection bias**, as clinicians with prior interest in or experience with clear aligner therapy may have been more likely to respond.

Certain inclusion criteria were set for the selection of the study population: orthodontists and oral and maxillofacial surgeons practicing in Saudi Arabia, regardless of gender, with or without previous experience in the use of clear aligners for orthognathic surgeries. Likewise, exclusion criteria were also set: practitioners unwilling to participate in the study, incomplete responses, and general dentists. A visual flowchart of the recruitment and inclusion/exclusion process is provided in Figure 1, illustrating the total number of professionals contacted, eligibility screening, and final response count. Given the achieved sample size, the results of inferential statistical analyses presented in this study should be interpreted as exploratory and hypothesis-generating rather than confirmatory.

**Figure 1.**
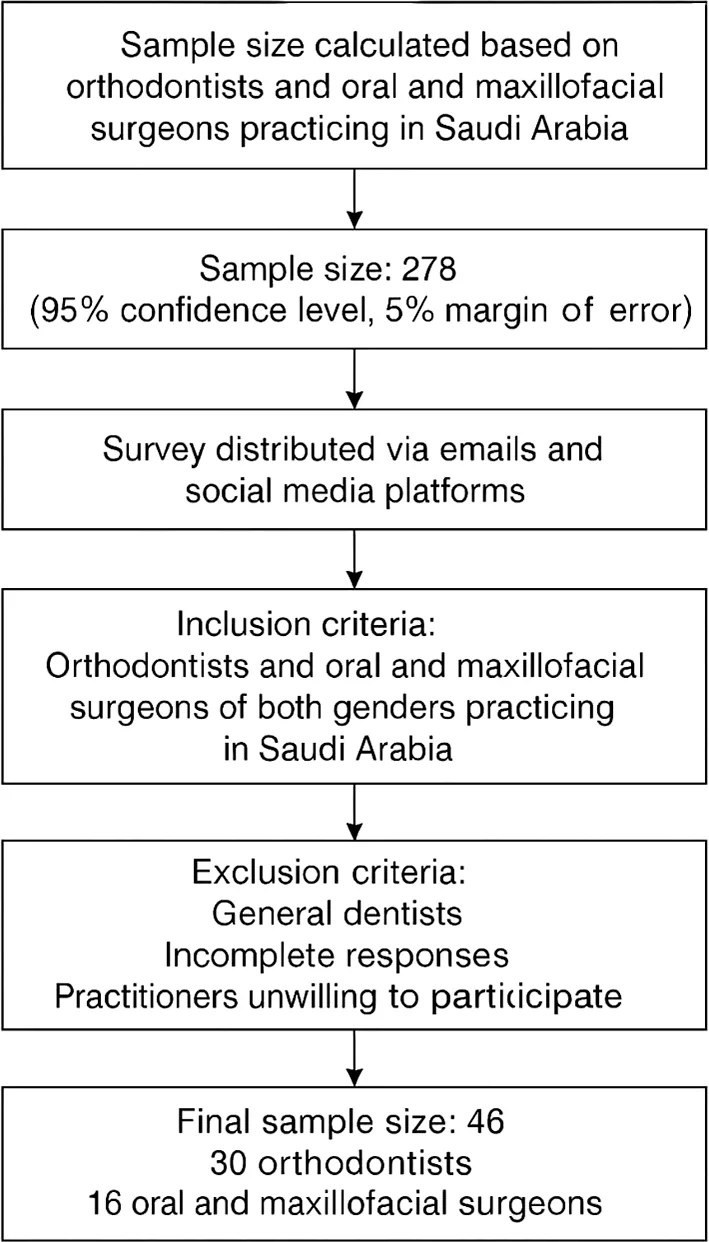
Flowchart of participant recruitment and selection process.

This figure illustrates the sampling method, survey distribution, inclusion and exclusion criteria, and the final sample of orthodontists and oral and maxillofacial surgeons who participated in the study.

Given the 16.5% response rate, potential non-response and self-selection biases were acknowledged. Low participation rates are common in clinician-based surveys due to professional workload and voluntary participation. To reduce bias, invitations were distributed through multiple channels (academic institutions, the Saudi Orthodontic Society, and social media), and anonymity was maintained to encourage honest responses. Non-parametric tests were applied to accommodate small-sample variability, and results were interpreted descriptively within an exploratory framework.

Although only 46 valid responses were obtained compared to the calculated minimum of 302, this reflects the limited pool of eligible specialists in Saudi Arabia. The small sample size reduces statistical power and increases the risk of Type II error; however, the study was designed as exploratory rather than inferential. Despite these limitations, the findings provide valuable pilot data on clinician awareness and readiness to integrate Clear Aligner Therapy (CAT) in orthognathic practice and establish a foundation for future multicenter research with adequate power and broader participation.

### Data collection instrument

The questionnaire comprised four sections. The first section includes demographic data. The second section was related to the current experience in orthognathic surgery and CAT. The third section addressed the knowledge of CAT used in orthognathic surgery patients. The fourth section was related to CAT practice in orthognathic surgery patients. To account for differences in expertise, the questionnaire included items assessing each respondent’s level of clinical involvement in orthognathic surgery cases such as the number of surgical cases managed per year, their role in interdisciplinary treatment planning, and prior use of CAT in such cases. Participants were also asked about their familiarity with digital and surgery-first workflows. These measures helped ensure that responses reflected informed perspectives and allowed subgroup comparisons based on clinical experience.

### Validity and reliability

The content validity of the questionnaire was assessed by two subject-matter experts an orthodontist and an oral and maxillofacial surgeon who evaluated each item for clarity, relevance, and alignment with the study objectives. Based on their feedback, minor linguistic modifications were made, and two items were reworded to eliminate ambiguity, particularly regarding post-surgical treatment steps. No items were removed, as all were deemed essential to the study scope. To assess reliability, the questionnaire was pilot-tested with 20 participants, who completed the survey twice, one week apart. Responses were compared using Cohen’s Kappa statistic, which demonstrated substantial agreement (κ > 0.70), confirming good test–retest reliability of the instrument.

### Statistical analysis

All the statistical analysis was performed using the Statistical Package for the Social Sciences Software (version 27, SPSS, Chicago, IL, USA).

A total of 50 responses were received, of which 4 were incomplete and excluded from the final analysis, leaving 46 valid responses for statistical evaluation. Descriptive statistics were used to summarize participant demographics, experience with orthognathic surgery and CAT, knowledge of CAT application, and related clinical practices. Given the limited sample size, the statistical analyses were primarily descriptive, and inferential tests were conducted for exploratory purposes only. Frequencies, percentages, means, and standard deviations (SD) were calculated for quantitative variables, while associations between categorical variables were tested using the chi-square test. Due to the small sample size, some expected cell counts may have been limited; therefore, these analyses were interpreted cautiously as exploratory indicators rather than confirmatory statistical evidence. Only fully completed questionnaires were retained to ensure data quality, and no imputation methods were applied due to the small dataset.

Prior to inferential testing, data normality was assessed using the Shapiro–Wilk test and inspection of histograms and Q–Q plots. The results indicated that the data were not normally distributed (p < 0.05), justifying the use of non-parametric tests. Accordingly, the Mann–Whitney U test was applied to compare knowledge levels between orthodontists and oral and maxillofacial surgeons using median knowledge scores derived from relevant questionnaire items. This approach was chosen over the independent samples t-test due to the non-normal distribution and small sample size. The analysis yielded a p-value of 0.59 indicated no statistically significant difference between the two groups.

A univariate linear regression analysis was performed to assess the relationship between demographic variables (age, gender, and professional experience) and the reported use of Clear Aligner Therapy (CAT) among orthodontists and oral and maxillofacial surgeons. The CAT usage variable was treated as a continuous composite score derived from questionnaire responses reflecting the frequency of CAT use in clinical practice. Model assumptions including linearity, independence, homoscedasticity, and normality of residuals were verified. The model produced an R ^2^ value of 0.38, suggesting that age accounted for approximately 38% of the observed variation in CAT usage within this dataset. Given the limited sample size and exploratory nature of the study, the regression results should be interpreted as hypothesis-generating observations rather than definitive predictors of CAT adoption.

This moderate explanatory power supports the model’s exploratory rather than predictive nature. Given the small sample size (n = 46), the analyses were interpreted cautiously. The limited number of responses may have reduced statistical power and increased the likelihood of Type II error—meaning that true associations could remain undetected. While no formal correction for multiple comparisons (e.g., Bonferroni adjustment) was applied, the results were interpreted cautiously because multiple statistical tests were conducted in a relatively small dataset. Accordingly, the study should be interpreted as an exploratory survey intended to provide preliminary insights into clinician perceptions of CAT in orthognathic surgery.

## Results

A total of 46 complete responses were obtained from 278 invitations, yielding a response rate of 16.5%. Given the limited sample size, the following results are presented primarily as descriptive observations. While this response rate is modest, it is within the range reported for similar surveys targeting specialized medical professionals. The study included 46 participants, predominantly male (67.4%) and aged mostly between 31-40 years (54.3%). Most participants had 6-15 years of professional experience (58.7%) and were mainly orthodontists (65.2%) or oral and maxillofacial surgeons (34.8%) (Supplementary Table S1). Among orthodontists (N = 30), 66.7% saw 1-4 orthognathic surgery patients monthly, and 56.7% practiced clear aligner therapy (CAT) with 1-10 patients weekly. However, 80% reported no prior experience using CAT in orthognathic surgery patients, indicating limited exposure to this treatment modality. However, 80% had never treated orthognathic surgery patients with CAT, indicating limited experience in this specific application (Supplementary Table S2). These findings should be interpreted within the context of the limited sample size, which may affect the reliability and external validity of the results. Detailed demographic and baseline characteristics are provided in **Supplementary Tables S1 and S2**.

Among oral surgeons (N = 16), half examined 5-10 orthognathic surgery patients weekly. About 44% treated 1-4 patients with CAT, while another 44% reported no CAT use, suggesting variable levels of clinical exposure to CAT among surgeons ( Table 1).

**Table 1.** The current experience of the oral and maxillofacial surgeons in orthognathic surgery and use of Clear Aligner Therapy (CAT) (N = 16).

Categories	Frequency range (No. of patients)	Frequency (n)	Percentage (%)	p-value
How often do you see orthognathic surgery patients in your practice weekly?	1-4	5	31.2	0.30 CI: (25.5, 74.5)
	5-10	8	50	
	>15	3	18.8	
How many orthognathic surgery patients have you treated using clear aligners (pre or post-surgery)	1-4	7	43.8	0.02 CI: (19.5, 68.1)
	5-10	1	6.2	
	>15	1	6.2	
	None	7	43.8	

Statistical test: Chi-square test; Abbreviations: CI, Confidence Interval; p, probability value.

Orthodontists expressed challenges primarily in pre-surgical alignment, leveling, and space closure, with 26.7%-36.7% agreeing on difficulties. Most agreed that malocclusion type influences the choice between CAT and fixed appliances (83.3%) ( Table 2).

**Table 2.** Knowledge of the orthodontists regarding CAT (N = 30).

Questions	Response (frequency (%))						
	Agree	Strongly agree	Disagree	Strongly disagree	Neutral	I don’t know	Not applicable
It is challenging to achieve the needed pre-surgical alignment required for accurate surgical setup	5 (16.7)	5 (16.7)	8 (26.7)	2 (6.7)	6 (20)	3 (10)	1 (3.3)
It is challenging to achieve the needed pre-surgical levelling to facilitate orthognathic surgical movements	7 (23.3)	1 (3.3)	7 (23.3)	2 (6.7)	8 (26.7)	4 (13.3)	1 (3.3)
It is challenging to achieve the needed pre surgical space closure necessary for orthognathic preparation	6 (20)	5 (16.7)	10 (33.3)	1 (3.3)	4 (13.3)	3 (10)	1 (3.3)
The type of malocclusion plays a role in the treatment choice (CAT vs fixed appliance) for surgical planning	8 (26.7)	17 (56.7)	0 (0)	1 (3.3)	2 (6.7)	1 (3.3)	1 (3.3)
CAT takes longer pre-surgical time compared with fixed appliances	4 (13.3)	3 (10)	8 (26.7)	1 (3.3)	11 (36.7)	1 (3.3)	2 (6.7)
CAT pre-surgical outcomes are similar to fixed appliance treatment	5 (16.7)	4 (13.3)	5 (16.7)	3 (10)	7 (23.3)	2 (6.7)	4 (13.3)
It is challenging to achieve the needed post-surgical refinement using CAT	5 (16.7)	3 (10)	7 (23.3)	2 (6.7)	7 (23.3)	4 (13.3)	2 (6.7)
It is challenging to obtain post-surgical occlusion and stability with CAT	10 (33.3)	3 (10)	8 (26.7)	1 (3.3)	3 (10)	3 (10)	2 (6.7)

Statistical test: Chi-square test; Abbreviations: CAT, Clear Aligner Therapy; p, probability value.

Oral surgeons highlighted challenges in surgical planning and intraoperative fixation, but many disagreed that final stable occlusion post-surgery was difficult to achieve ( Table 3).

**Table 3.** Knowledge of the oral and maxillofacial surgeons regarding CAT (N = 16).

Questions	Response (frequency (%))					
	Agree	Strongly agree	Disagree	Strongly disagree	Neutral	I don’t know
It is challenging to perform the surgical planning in CAT	4 (25)	1 (6.2)	2 (12.5)	3 (18.8)	5 (31.2)	1 (6.2)
It is challenging to achieve intraoperative intermaxillary fixation (IMF) in CAT	5 (31.2)	3 (18.8)	3 (18.8)	2 (12.5)	2 (12.5)	1 (6.2)
It is challenging to use the interim splint during the surgical procedure in CAT	3 (18.8)	2 (12.5)	2 (12.5)	3 (18.8)	5 (31.2)	1 (6.2)
It is challenging to obtain a final stable occlusion after the surgery	3 (18.8)	2 (12.5)	6 (37.5)	1 (6.2)	4 (25)	0 (0)
CAT can be used with segmental osteotomies procedures	5 (31.2)	3 (18.8)	3 (18.8)	3 (18.8)	1 (6.2)	1 (6.2)
CAT can be used with excessive maxillary or mandibular movements (advancement and setback)	6 (37.5)	4 (25)	1 (6.2)	3 (18.8)	1 (6.2)	1 (6.2)
CAT can be used with excessive rotational movements	7 (43.8)	1 (6.2)	2 (12.5)	2 (12.5)	3 (18.8)	1 (6.2)
CAT can be used in surgery first concept	7 (43.8)	3 (18.8)	1 (6.2)	1 (6.2)	3 (18.8)	1 (6.2)
CAT can be used in mandible first concept	6 (37.5)	2 (12.5)	3 (18.8)	1 (6.2)	3 (18.8)	1 (6.2)

Statistical test: Chi-square test; Abbreviations: CAT, Clear Aligner Therapy; IMF, Intermaxillary Fixation; CI, Confidence Interval; p, probability value.

No significant difference in overall knowledge of CAT between orthodontists and oral surgeons was found (p = 0.59), suggesting comparable expertise levels ( Table 4).

**Table 4.** Comparison of average knowledge scores between orthodontists and oral surgeons (Mann-Whitney U test).

Question item	Orthodontists (Mean score) CI: (3.42, 4.26)	Oral surgeons (Mean score) CI: (3.95, 4.48)	p-value
Q1	3.72	3.56	0.59 (3.42, 4.26)
Q2	3.48	4.25	
Q3	3.97	4.44	
Q4	4.86	3.94	
Q5	3.41	4.31	
Q6	3.65	4.5	
Q7	3.39	4.25	
Q8	4.25	4.44	
Q9		4.25	

Statistical test: Mann–Whitney U test; Abbreviations: CAT, Clear Aligner Therapy; CI, Confidence Interval; p, probability value.

Post-surgical management commonly required fixed appliances and temporary anchorage devices (TADs), but only 6.7% felt fully confident in using CAT in orthognathic cases ( Table 5).

**Table 5.** Responses of study orthodontists CAT practice in orthognathic surgery patients (N = 30).

Question	Responses (frequency (%))						
	Always	Often	Sometimes	Never	Rarely	I don’t know	Not Applicable
The case complexity drive your decision in selecting CAT as a treatment choice	18 (60)	4 (13.3)	4 (13.3)	1 (3.3)	1 (3.3)	1 (3.3)	1 (3.3)
The availability of the needed digital tools affect your decision in selecting CAT as a treatment of choice	8 (26.7)	6 (20)	9 (30)	3 (10)	2 (6.7)	1 (3.3)	1 (3.3)
The cost of the treatment plays a major role in selecting CAT as a treatment choice	10 (33.3)	7 (23.3)	9 (30)	2 (6.7)	0 (0)	1 (3.3)	1 (3.3)
The patient demands drive your decision in selecting CAT as a treatment choice	8 (26.7)	10 (33.3)	8 (26.7)	0 (0)	1 (3.3)	2 (6.7)	1 (3.3)
The skills of the maxillofacial surgeon drive your decision in selecting CAT as a treatment choice	5 (16.7)	8 (26.7)	8 (26.7)	4 (13.3)	2 (6.7)	1 (3.3)	1 (3.3)
The cooperation of the maxillofacial surgeon drive your decision in selecting CAT as a treatment choice	9 (30)	7 (23.3)	8 (26.7)	2 (6.7)	1 (3.3)	1 (3.3)	2 (6.7)
The patient cooperation affect your decision in selecting CAT as a treatment (before or after surgery)	16 (53.3)	10 (33.3)	1 (3.3)	1 (3.3)	0 (0)	1 (3.3)	1 (3.3)
The reduced time of the treatment plays a major role in selecting CAT as a treatment choice	9 (30)	5 (16.7)	9 (30)	3 (10)	2 (6.7)	1 (3.3)	1 (3.3)
The reduced number of visits of the treatment plays a major role in selecting CAT as a treatment choice	8 (26.7)	5 (16.7)	10 (33.3)	3 (10)	2 (6.7)	1 (3.3)	1 (3.3)
The reduced pain during treatment plays a major role in selecting CAT as a treatment choice	4 (13.3)	3 (10)	9 (30)	6 (20)	5 (16.7)	1 (3.3)	2 (6.7)
CAT combined with fixed appliances is required before the surgery	5 (16.7)	4 (13.3)	14 (46.7)	2 (6.7)	2 (6.7)	2 (6.7)	1 (3.3)
Fixed appliance bonding is needed for post-surgical management	4 (13.3)	7 (23.3)	16 (53.3)	0 (0)	1 (3.3)	1 (3.3)	1 (3.3)
Temporary anchorage device (TADS) are needed for post-surgical management	0 (0)	5 (16.7)	18 (60)	0 (0)	5 (16.7)	1 (3.3)	1 (3.3)
You are confident of using CAT in orthognathic patients	2 (6.7)	4 (13.3)	13 (43.3)	3 (10)	3 (10)	4 (13.3)	1 (3.3)

Statistical test: Chi-square test; Abbreviations: CAT, Clear Aligner Therapy; TADs, Temporary Anchorage Devices; p, probability value.

Fixed appliances were often requested pre-surgery (43.8%), and intraoperative fixation relied mostly on IMF screws (37.5%) ( Table 6).

**Table 6.** Responses of study oral and maxillofacial surgeons CAT practice in orthognathic surgery patients (N = 16).

Question	Responses (frequency (%))						
	Always	Often	Sometimes	Never	Rarely	I don’t know	Not applicable
The surgery time is increased in CAT cases	1 (6.2)	2 (12.5)	3 (18.8)	3 (18.8)	5 (31.2)	2 (12.5)	0 (0)
It is difficult to control the post-operative occlusion on CAT patients	2 (12.5)	2 (12.5)	8 (50)	1 (6.2)	1 (6.2)	2 (12.5)	0 (0)
How frequent you ask the orthodontist to bond fixed appliances before the surgery (if the pre-surgical orthodpntics was using clear aligner)	7 (43.8)	5 (31.2)	1 (6.2)	1 (6.2)	0 (0)	**1 (6.2)**	**1 (6.2)**
How frequent do you rely on IMF screws for intraoperative IMF	6 (37.5)	3 (18.8)	3 (18.8)	1 (6.2)	3 (18.8)	0 (0)	0 (0)
How frequent do you rely on TADS intraoperative IM	2 (12.5)	2 (12.5)	1 (6.2)	5 (31.2)	5 (31.2)	1 (6.2)	0 (0)
How frequent do you rely on archbars intraoperative IMF	1 (6.2)	3 (18.8)	2 (12.5)	5 (31.2)	4 (25)	1 (6.2)	0 (0)
The post-operative occlusion for CAT cases is guided using elastics	2 (12.5)	3 (18.8)	4 (25)	1 (6.2)	4 (25)	1 (6.2)	1 (6.2)
The post-operative occlusion for CAT is guided using wires	1 (6.2)	1 (6.2)	3 (18.8)	4 (25)	3 (18.8)	3 (18.8)	1 (6.2)
How frequent do you rely on TADS to manage post-operative occlusion relapse	1 (6.2)	3 (18.8)	4 (25)	5 (31.2)	0 (0)	2 (12.5)	1 (6.2)
How frequent do you rely on arch bars to manage post-operative relapse	2 (12.5)	1 (6.2)	2 (12.5)	7 (43.8)	2 (12.5)	1 (6.2)	1 (6.2)
How frequent do you rely on orthodontic fixed appliance to manage post-operative relapse	6 (37.5)	1 (6.2)	5 (31.2)	2 (12.5)	2 (12.5)	0 (0)	0 (0)

Statistical test: Chi-square test; Abbreviations: CAT, Clear Aligner Therapy; IMF, Intermaxillary Fixation; TADs, Temporary Anchorage Devices; p, probability value.

Age was the only demographic factor significantly correlated with CAT usage among both orthodontists (p = 0.01, adjusted R ^2^ = 0.284) and oral surgeons (p = 0.02, adjusted R ^2^ = 0.25). Gender and years of experience showed no significant correlation ( Table 7).

**Table 7.** Univariate linear regression model for correlation between CAT usage and demographical parameters.

Variable	*R ^2^*	*Adjusted R ^2^*	P-value
**Orthodontists**			
Gender	0.021	-0.014	0.44
Age	0.383	0.284	0.01
Experience	0.154	0.019	0.361
**Oral and maxillofacial surgeons**			
Gender	0.05	-0.01	0.38
Age	0.30	0.25	0.02
Experience	0.13	0.07	0.15

Statistical test: Univariate linear regression analysis; Abbreviations: CAT, Clear Aligner Therapy; R ^2^, Coefficient of Determination; p, probability value.

## Discussion

### Overview of findings

While traditional fixed appliances have long been the standard in pre and post-surgical orthodontic treatment, there is growing interest in the application of clear aligners, especially given their aesthetic appeal and enhanced patient comfort. However, the survey revealed a range of opinions on the suitability of CAT for managing complex skeletal discrepancies often requiring surgical intervention. This study offers exploratory insights into how Saudi orthodontists and oral and maxillofacial surgeons perceive the integration of CAT in orthognathic cases.

Out of the professionals surveyed, 46 responded, yielding a response rate of 16.5%. Respondents represented diverse regions and included proportionate representation of both orthodontists and oral surgeons. The results revealed general awareness of CAT and its emerging role in orthognathic cases within the surveyed Saudi sample, while also highlighting uncertainty regarding its practical application. The Mann-Whitney U test did not detect a statistically significant difference in overall CAT knowledge between orthodontists and surgeons (p = 0.59). However, given the limited sample size, this observation should be interpreted cautiously and considered hypothesis-generating rather than conclusive.

While the response rate was modest, it aligns with comparable clinician-based surveys internationally, where participation rates commonly range between 15–25%. Nevertheless, the low response rate may introduce non-response bias, as clinicians with prior interest or familiarity with clear aligner therapy may have been more inclined to participate. ^20–22^ Despite this, the responses provide valuable preliminary insights into clinician perspectives within Saudi Arabia, a region where such data remain limited.

### Knowledge and attitudes toward CAT

The findings of the present study highlight several clinically relevant themes regarding CAT use in orthognathic cases. A significant proportion of respondents reported challenges with pre-operative positioning, leveling, and space-closing with CAT. Most agreed that the type of malocclusion affects the choice between CAT and a fixed appliance, with opinions divided on whether CAT outcomes are comparable to fixed appliances, particularly in post-surgical occlusion. These findings may suggest that Saudi clinicians recognize the potential of CAT but remain cautious regarding its predictability in complex cases. Similar caution has been reported globally, with clinicians citing limitations in root control, torque, and vertical adjustments. ^20^^,^^21^ Systematic reviews suggest that while CAT is increasingly effective for mild-to-moderate malocclusions, evidence for its use in surgical orthodontics remains limited. ^23–25^

Regional variation in responses may reflect differences in population density, clinical training, and access to CAT systems. In South Korea, where computer-assisted orthognathic surgery (CAOS) is more established, clinicians report more advanced integration of CAT into surgery-first workflows. However, even there, broader clinical use is constrained by cost, the need for validation, and specialized training. ^22^ Globally, while enthusiasm for CAT is rising, systematic reviews suggest continued caution in surgical applications due to unresolved concerns about long-term stability, workflow complexity, and lack of standardized protocols. ^26, 27^

The absence of significant knowledge differences between orthodontists and surgeons aligns with literature showing both groups have similar exposure to CAT protocols, especially in digitally supported workflows like 3D planning and simulation. ^28^

These observations should be interpreted cautiously because the exploratory nature of the study and the limited sample size restrict the ability to generalize the findings to the broader clinician population.

### Comparative knowledge across specialties

This reflects global literature showing that both groups have similar exposure to digital workflows, particularly where interdisciplinary planning is emphasized. ^29^ According to published literature, while orthodontists and oral and maxillofacial surgeons agree on the benefits of CAT particularly for patient esthetic and comfort they also remain critical of the stability and control during IMF, as well as the accuracy of postoperative occlusion. ^7, 27^ Particularly in surgery, first workup, digital planning along with coordination of appliances are challenging and require greater interdisciplinary collaboration. ^28^

Local clinicians have expressed high confidence in static CAOS tools for CAT, but have only limited adaptability to the full integration of such systems because of the cost and education base. ^22^ Long-term evidence and universal protocols remain insufficient, reinforcing the need for cautious optimism. ^27^

### Awareness across populations

The awareness and perception of health professional graduates regarding the use of clear aligners in orthodontics ranged from moderate to low. ^30^ Furthermore, several other studies have evaluated awareness of clear aligners among various groups, including dentists, dental graduates, the general public, and orthodontists. For instance, a study in Saudi Arabia examined dentists’ knowledge of clear aligners, revealing moderate awareness, with most respondents not opting for this treatment option for their patients. ^31^ Another study performed in India, found that 93.5% of dental interns and 83.6% of dental undergraduates were aware of clear aligners, ^32^ while another study was performed in Saudi Arabia and reported that only 19.6% of the general public, out of 934 respondents, were aware of clear aligners. ^33^

CAT is increasingly adopted in routine orthodontic practice; however, its application in orthognathic surgery remains limited, as also reflected in the present study findings.

### Attitudes toward CAT vs fixed appliances

A study performed in Canada and almost half of the orthodontists (47%) combined CAT with fixed appliances. ^34^

Across regions, CAT is generally perceived as more comfortable and aesthetically appealing than traditional fixed appliances. In East Asia, for example, social media influence, digital marketing, and cultural emphasis on facial harmony contribute to higher aligner demand, especially among younger adults. ^35^

In areas with an emphasis on precision of the treatment (e.g., parts of Europe and North America), clinicians continue to be skeptical of CAT’s capacity to address complex orthodontic mechanics. This concern is especially prevalent in the area of surgery, where clinicians expect outcomes to be highly predictable, and are slow to embrace aligners in the absence of clearly defined protocols and long-term success rates. Here, traditional braces remain more common due to their lower cost and widespread familiarity.

### Demographic trends in usage

This may be attributed to greater exposure to digital workflows and CAT protocols during training. Increased comfort with new technologies, and responsiveness to patient demand for esthetic and metal-free treatments. These clinicians are especially confident using CAT in mild to moderate adult malocclusions, where case complexity remains manageable. ^20^

Conversely, in parts of the Middle East and Asia, CAT use is more common among experienced clinicians. Here, senior practitioners often dominate surgical orthodontics, and their adoption of CAT is shaped more by clinical judgment and accumulated experience than by early exposure during training. Still, adoption among younger practitioners is rising, driven by evolving patient expectations, improved curriculum, and broader access to aligner systems.

Across all regions, adults remain the primary demographic for CAT, particularly working professionals who prioritize aesthetics and convenience. Studies also highlight increasing use of CAT in adolescents and even children for dentoalveolar changes, although evidence for skeletal correction is less conclusive. ^36^

Clinician experience also directly affects treatment outcomes: those using structured case selection protocols or assessment tools like the CAT-CAT index report improved predictability and fewer refinements. ^37^ Emerging technologies such as machine learning models that predict treatment refinement needs are poised to further assist both novice and experienced practitioners in case planning. ^38^

Socioeconomic factors also influence usage trends. In high-income countries, access to advanced 3D printing and aligner software enhances usage across age groups. However, in low-resource settings, cost and access barriers can limit the use of aligners, even among trained professionals. Multinational providers and mobile digital scanning platforms have helped expand aligner availability globally, yet disparities in access remain, particularly in underserved or rural regions.

Because the statistical analyses were exploratory and conducted on a limited sample, the observed association between age and CAT usage should be interpreted as a hypothesis-generating observation requiring confirmation in larger studies.

### Regional and global public/Professional awareness

Our study’s findings regarding professional awareness among Saudi orthodontists and oral surgeons appear to align with the limited public awareness reported in prior regional research. A recent study by Alsaeed et al. (2023) reported that only 19.6% of the general Saudi public were aware of clear aligner options for orthodontic care. ^12^ This figure contrasts with awareness levels among Indian dental interns, which were as high as 93.5%, suggesting that educational exposure during undergraduate training may significantly enhance familiarity and confidence in CAT.

This discrepancy underscores a potential gap between rising aesthetic-driven patient demand and limited public awareness. Unlike in digitally-saturated markets, consumers may be less likely to proactively seek CAT unless informed by a clinician, suggesting a need for targeted public education campaigns and greater integration of CAT discussions during consultations. Bridging this gap may require collaboration between dental professionals, academic institutions, and aligner manufacturers to promote evidence-based awareness and ensure patients receive comprehensive treatment information.

### Clinical challenges and workflow uncertainties

Respondents in the present study did not have a clear stance regarding whether CAT takes longer pre-surgical time or not. They believe CAT pre-surgical outcomes are similar to those of fixed appliance treatment. Respondents’ uncertainty regarding whether CAT requires more time before surgery. This may be attributed to differences in the complexity of the case and the treatment goals of the patient. Compared to fixed appliances that have a long history of treating a wide variety of complex malocclusions, CAT is still new, and its effectiveness may vary considering the malocclusion type, requirement in tooth movement, and the orthodontist’s experience in using technology. ^39^

A few clinicians stated that CAT can lead to pre-surgical lag because of the repetitive modifications of digital treatment plans, or challenges associated with achieving final tooth position where aligner refinements are required. Moreover, patient comfort and compliance in terms of aligner wear, which can vary widely, can affect these time perceptions. ^7, 10, 11^

In our Saudi orthodontists and oral maxillofacial survey, many of the respondents expressed anxiousness about workflow interference which has been blocking the best integration of the CAT with the orthognathic protocols. Critical factors were pre-surgical treatment delay, phase II with multiple aligner fits and aligner mechanics’ limitations in masking complex skeletal discrepancies.

These results confirm the observations of international literature where it is underlined how contemporary CAT systems are frequently unable to make sufficiently complex orthognathic cases without the adoption of hybrid protocols. For instance, 3D digital planning workflows integrated with computer-aided technology (CAT) custom titanium plates have been performed by North American surgeons has improved surgical predictability and postoperative management. ^28^ Although promising, these approaches need dedicated software, additional coordination at the chairside, and higher costs, which diminishes their applicability under low-resource settings.

In Europe, similar challenges are addressed through adjunctive techniques, such as temporary anchorage devices (TADs) and segmental surgery, to supplement CAT in treating skeletal Class III deformities and asymmetries, especially in surgery-first approaches. ^26, 28^

Our findings appear to align with international observations suggesting that CAT may represent a promising but technically challenging modality that demands interdisciplinary teamwork, strong digital infrastructure and flexible case-by-case planning.

### Practice preferences and treatment decision factors

The current study highlights that clinicians in Saudi Arabia prioritize case complexity, patient demand, and comfort when selecting between Clear Aligner Therapy (CAT) and traditional fixed appliances. Survey responses showed that esthetic-driven demand, particularly among younger adults, plays a growing role in treatment planning. Nevertheless, clinicians remained cautious about using CAT in complex skeletal cases, especially those requiring precise post-surgical occlusion and long-term stability.

Although final refinements post-surgery were not widely reported as problematic, achieving ideal occlusion remains a key concern. Factors such as patient cooperation, ^8^ treatment cost and duration, ^9^ and coordination with oral surgeons often influence treatment selection. Some clinicians opt for hybrid protocols, combining CAT with fixed appliances or adjunctive tools like Temporary Anchorage Devices (TADs) in post-surgical phases. ^7^

Skepticism toward CAT for skeletal discrepancies stems from its original design for dental malocclusions rather than orthognathic correction. ^1^ Limitations in tooth movement control, especially without mid-course corrections, and short-term clinical experience further compound uncertainty. ^10^

Globally, similar patterns emerge. In Europe, clinicians often adapt to patient demands for removable appliances even in less complex cases, balancing esthetic preferences with biomechanical viability. ^40, 41^ In contrast, Asian clinicians emphasize predictability and long-term outcomes, favoring fixed or hybrid options for skeletal corrections. ^22, 28^ These regional nuances underscore that CAT adoption is shaped by clinical culture, economic context, and patient expectations. ^35^

It is important to note that the convenience sampling strategy and relatively small number of respondents may limit the generalizability of these findings to the broader population of orthodontists and oral and maxillofacial surgeons in Saudi Arabia. Future studies with larger, multicenter samples are required to confirm these observations.

### Implications for clinical protocols and training

The results of this investigation highlight an urgent need for evidence-based, standardized clinical protocols for the application of Clear Aligner Therapy (CAT) in orthognathic procedures. The present clinical ambiguities, particularly case selection, treatment sequence, digital treatment planning, and appliance modification in surgery-first protocols, reveal notable deficiencies in defined criteria and predictable results.

And this gap is not specific locally. Others overseas report similar struggles, and in places like North America and Europe, hybrid models of providing care are slowly starting to take off. These commonly incorporate 3D surgical planning, Temporary Anchorage Devices (TADs) and segmental surgeries to enhance predictability, stability and control for CAT-based interventions. ^28^ Even though there has been progress on this field, the lack of standardized protocols is still a big limitation; and especially for more complex skeletal deformities. Overcoming these implementation barriers will require major training reforms. In light of the study findings, several practical and educational recommendations can be drawn.

For orthodontists, postgraduate and continuing education programs should include structured modules on digital workflows, aligner biomechanics, case selection, and integration of hybrid treatment strategies when CAT alone may be insufficient. These competencies are essential to improve confidence and predictability in managing complex surgical orthodontic cases.

For oral and maxillofacial surgeons, the training focus should be different—centered on understanding the surgical coordination aspects of CAT, including splint adaptation, intermaxillary fixation (IMF), and postoperative elastic management. Rather than orthodontic biomechanics, surgeons primarily need awareness of CAT’s surgical limitations and its implications for occlusal stability and intraoperative workflow.

To translate these findings into practice, two key recommendations can be proposed:

1. Educational integration: Incorporation of CAT and digital workflow training into orthodontic and surgical training programs, supported by structured courses and continuing professional development initiatives.
2. Interdisciplinary and research collaboration: Promotion of collaborative case planning through interdisciplinary workshops, alongside multicenter and international research efforts to develop standardized, evidence-based clinical protocols.

These strategies would help standardize CAT use in surgical orthodontics, enhance interdisciplinary coordination, and align training in Saudi Arabia with international standards for digitally driven, patient-centered care.

Overall, the findings of this study should be considered preliminary and serve primarily to highlight areas requiring further investigation in larger, adequately powered studies.

### Limitations

Despite providing valuable insights into clinician perspectives on clear aligner therapy (CAT) in orthognathic surgery, this study is not without limitations.

First, the sample size was relatively small, with only 46 responses obtained out of 278 invitations, resulting in a response rate of 16.5%. While this rate is comparable to similar professional surveys, it is substantially lower than the calculated minimum required sample size (n = 302) and therefore limits the statistical power of the study. This increases the risk of both Type II error (failure to detect true associations) and Type I error (false-positive findings due to multiple testing), and restricts the generalizability of the findings across the broader population of orthodontists and surgeons in Saudi Arabia.

The low participation rate also raises the possibility of non-response bias (selection bias), where clinicians more familiar with or interested in CAT may have been more likely to respond. Although a formal non-responder analysis could not be conducted due to participant anonymity, this potential self-selection bias should be acknowledged, as it may have led to an overrepresentation of clinicians with prior interest or familiarity with CAT.

Given the limited sample size, the statistical analyses—including chi-square testing and univariate regression—should be interpreted strictly as exploratory. Although the regression model demonstrated a moderate R ^2^ value (0.38), this finding should be considered hypothesis-generating rather than confirmatory. Given the limited sample size, the statistical analyses—including chi-square testing and univariate regression—should be interpreted strictly as exploratory. Although the regression model demonstrated a moderate R ^2^ value (0.38), this finding should be considered hypothesis-generating rather than confirmatory. Future studies with larger, randomized samples are necessary to confirm and expand upon these findings. The study employed a non-probability convenience sampling approach, which, while pragmatic and commonly used in exploratory healthcare research, limits the representativeness of the sample and further constrains external validity. This constraint may affect the generalizability of findings to the entire population of orthodontists and oral and maxillofacial surgeons in Saudi Arabia. limits the representativeness of the sample and further constrains external validity.

Second, while the study draws on international comparisons to contextualize findings, these should be interpreted with caution. Variations in aligner systems (e.g., Invisalign, Clarity, AngelAlign), digital infrastructure, and regulatory environments can impact workflows, confidence, and adoption, complicating direct cross-country comparisons.

Third, the study relied on self-reported data, which may be subject to recall bias or overestimation of familiarity and competence with CAT protocols. This is particularly relevant in areas where structured CAT training is lacking or inconsistently delivered.

Despite these limitations, the study adds to the global understanding of CAT implementation and highlights important educational and clinical gaps that can be addressed in future research and policy reforms.

Given the complex, multidisciplinary nature of surgical orthodontics, ongoing collaboration and mutual learning between orthodontists and surgeons are essential to address evolving clinical expectations. Literature increasingly supports collaborative CAT planning, especially for protocols such as surgery-first, which require coordinated efforts in digital planning, appliance design, and clinical workflow. ^28^ A standardized protocol and education effort may help mitigate current clinical uncertainties and enhance patient’s outcomes. The establishment of a standardized clinical protocol and a consolidated training program may be an approach to overcome these inconsistencies and improve the prognosis of patients undergoing complex surgical procedures.

## Conclusion

This exploratory survey highlights limited clinical experience with clear aligner therapy (CAT) in orthognathic surgery among Saudi orthodontists and oral and maxillofacial surgeons, along with concerns regarding its predictability and surgical application. Clinicians reported challenges in both orthodontic phases (e.g., pre-surgical alignment) and surgical procedures, including intraoperative fixation, splint use, and intermaxillary elastics.

Given that the achieved sample size (n = 46) was substantially lower than the calculated requirement (n = 302), the findings should be interpreted as preliminary. Further large-scale, multicenter studies are needed to validate these observations and support the development of standardized protocols for CAT-assisted orthognathic treatment.

## Underlying data

**Repository name:** The Use of Clear Aligner Therapy for Orthognathic Surgery Patients: A Cross-Sectional Survey Among Saudi Orthodontics and Oral and Maxillofacial Surgeons. https://doi.org/10.5281/zenodo.16977338

The project contains the following underlying data:

FINAL The use of clear aligner therapy for orthognathic surgery patients (Responses)-3.xlsx (raw).

Supplementary Tables.docx **(Tables).**

Data are available under the terms of the Creative Commons Zero “No rights reserved” data waiver (CC0 1.0 Public domain dedication).

## Software availability

The statistical analysis for this study was performed using the Statistical Package for the Social Sciences (SPSS), version 27 (SPSS Inc., Chicago, IL, USA). The software used for digital treatment planning and data collection, including the Google Forms platform for survey distribution, are freely available for public use. No specialized or proprietary software was required for the completion of this study beyond those mentioned above.

## Supplementary materials

The complete survey questionnaire used in this study is provided in *Supplementary Table S1.* It includes all sections used to assess demographics, clinical experience, knowledge, and attitudes toward the use of clear aligner therapy (CAT) in orthognathic surgery. An anonymized dataset of all participant responses (n = 46), excluding any personal identifiers, is provided as *Supplementary Table S2* to support transparency and allow for further secondary analysis.
