# The Use of Clear Aligner Therapy for Orthognathic Surgery Patients: A Cross-Sectional Survey Among Saudi Orthodontists and Oral and Maxillofacial Surgeons

**DOI:** 10.12688/f1000research.170095.5

**Published:** 2026-10-14

**Authors:** Ahmad Salem Assari, Muslat A Bin Rubaia’an, Diaa Taisir Al-Marhoun, Hayat Arafah, AlAnood N Bin Saedan, Hajar Dar Salamah, Raneem Mohammed Aljomiey

**Affiliations:** 1 Department of Oral and Maxillofacial Surgery and Diagnostic Sciences, College of Medicine and Dentistry, Riyadh Elm University, Riyadh, Riyadh Province, Saudi Arabia; 2 Department of Dentistry, King Faisal Specialist Hospital and Research Centre, Riyadh, Saudi Arabia; 3 Department of Maxillofacial Surgery, Prince Sultan Military Medical City, Riyadh, Riyadh Province, Saudi Arabia; 4 Department of Restorative Dentistry, College of Medicine and Dentistry, Riyadh Elm University, Riyadh, Saudi Arabia; 5 Alwattan medical complex 3, Riyadh, Saudi Arabia; 6 Department of Preventive Dentistry, College of Medicine and Dentistry, Riyadh Elm University, Riyadh, Saudi Arabia

**Keywords:** Orthognathic surgery, Clear aligner therapy (CAT), Orthodontics, Oral and maxillofacial surgery, Saudi Arabia, Surgical orthodontics

## Abstract

**Background:**

Orthognathic surgery corrects severe skeletal discrepancies that cannot be addressed by orthodontics alone. With advancements in digital workflows and CAD/CAM technologies, clear aligner therapy (CAT) has emerged as a potential adjunct in surgical cases. However, its clinical application remains under-researched. This study aimed to exploratorily assess the knowledge, clinical experience, and attitudes of orthodontists and oral and maxillofacial surgeons in Saudi Arabia regarding CAT in orthognathic surgery patients.

**Methods:**

A cross-sectional survey was conducted from April 1 to May 31, 2023, using a closed-ended online questionnaire distributed to specialists across Saudi Arabia. The survey gathered demographic and clinical data. Statistical analysis included descriptive methods, chi-square testing, and univariate regression. Given the limited sample size, inferential analyses were performed for exploratory purposes and interpreted cautiously.

**Results:**

Out of 278 invited participants, 46 complete responses (16.5%) were received, which was substantially lower than the calculated minimum sample size. Most participants were aged 31–40 years and had 6–10 years of experience. Orthodontists accounted for 65.2% of the respondents, with oral and maxillofacial surgeons at 34.8%. While most treated 1–4 orthognathic patients monthly, 80% had no experience using CAT in these cases Although CAT was commonly used in routine orthodontic practice, its application in orthognathic surgery cases was limited. Exploratory regression analysis suggested a possible association between clinician age and CAT usage (R ^2^ = 0.38), whereas gender and years of experience did not show clear associations.

**Conclusion:**

This exploratory survey provides preliminary insight into current clinician experience and perceptions regarding the use of clear aligner therapy in orthognathic surgery in Saudi Arabia. Given the low response rate and limited sample size, the findings should be interpreted cautiously as descriptive and hypothesis-generating rather than confirmatory, and larger multicenter studies are needed before any definitive conclusions can be drawn.

## Introduction

Orthognathic surgery remains the treatment of choice for adults with severe dentofacial deformities or skeletal discrepancies that cannot be corrected by orthodontic camouflage alone. ^1, 2^ Successful outcomes require a coordinated interdisciplinary approach involving both orthodontists and oral and maxillofacial surgeons. ^1, 2^

Orthodontic management for surgical cases traditionally involves pre-surgical decompensation using fixed appliances followed by post-surgical refinement. This study primarily refers to the conventional orthodontics-first approach, where pre-surgical orthodontic preparation is essential prior to orthognathic surgery. Although surgery-first protocols have been introduced in recent years, they eliminate or reduce the need for extensive pre-surgical orthodontics and therefore involve different clinical considerations. As such, the present investigation focuses on clinician perspectives within the orthodontics-first framework, where the role of clear aligner therapy in pre- and post-surgical phases remains clinically relevant. Fixed appliances, typically metal or ceramic brackets connected by archwires, remain the gold standard in this context due to their ability to deliver controlled tooth movements in all three planes of space. ^3, 4^ However, growing aesthetic demands, changing patient preferences, and concerns about oral hygiene and periodontal health have contributed to decreased patient acceptance of fixed appliances. ^5– 7^

The introduction of clear aligner therapy (CAT) in the late 20th century has provided an alternative, especially for patients prioritizing aesthetics and comfort. ^8^ Initially indicated for mild to moderate malocclusions, CAT has evolved considerably with advances in digital imaging, 3D printing, and biomechanical modeling, allowing treatment of increasingly complex dental movements. ^1, 6, 9– 11^ CAT now features transparent, removable trays generated through digital treatment planning, offering improved aesthetics, patient comfort, oral hygiene, and reduced chair time. ^7, 8^ Its use in combination with surgery is emerging; however, its role differs between orthodontics-first and surgery-first protocols. In orthodontics-first approaches, CAT may be used in pre- and post-surgical phases, whereas in surgery-first protocols its role is more limited and evolving. ^7^

Meanwhile, in Saudi Arabia, the demand for clear aligners has risen in tandem with global trends as patients increasingly seek orthodontic solutions that are less conspicuous and more comfortable than traditional braces. ^12^ However, there is little evidence exploring clinicians’ readiness, experience, or confidence in integrating CAT into surgical orthodontics. This gap in empirical data limits understanding of how practitioners perceive its feasibility in complex surgical workflows. ^13^^,^^14^ This knowledge gap presents a barrier for both orthodontists and oral and maxillofacial surgeons in utilizing CAT effectively for complex skeletal discrepancies. Region-specific studies indicate that orthognathic surgery is among the most frequently performed oral and maxillofacial procedures in Saudi teaching hospitals, ^15^ and patient motivation is often driven by aesthetic concerns, with satisfaction rates exceeding 90%. ^16, 17^ Moreover, research highlights anatomical variations between regions within the country such as greater microgenia and longer facial profiles in southern populations which further emphasizes the need for personalized surgical planning and broader adoption of advanced, adaptable technologies like CAT. ^13, 18^

Given the rising popularity of CAT and the evolving landscape of orthodontic practice, there is a need to evaluate clinician perspectives in surgical contexts. The current study aimed to assess the knowledge, clinical experience, attitudes, and practices of Saudi orthodontists and oral and maxillofacial surgeons regarding the use of clear aligner therapy in orthognathic surgery patients.

## Methodology

### Study design, setting and ethical consideration

This cross-sectional study was ethically approved by the Institutional Review Board (IRB) of Riyadh Elm University (Approval No. [FUGRP/2023/303/929]) and conducted in full accordance with the Declaration of Helsinki. All participants received detailed information about the study objectives, procedures, and data confidentiality, and provided electronic informed consent prior to participation. Participation was voluntary, and anonymity and data confidentiality were maintained throughout.

A closed-ended, self-administered questionnaire was designed online using Google Forms and distributed among orthodontists and maxillofacial surgeons practicing in Saudi Arabia. The survey was conducted between April 1 and May 31, 2023. Participants were recruited through professional email lists, social media platforms, and direct distribution via professional organizations, including the Saudi Orthodontic Society and regional oral and maxillofacial surgery networks. To minimize sampling bias, invitations were sent broadly to licensed practitioners across academic, private, and governmental sectors.

### Sample size and population

According to recent data from the Saudi Commission for Health Specialties and regional workforce reports, there are an estimated 900–1,100 orthodontists and 400–500 oral and maxillofacial surgeons actively practicing in Saudi Arabia. ^19^ Our sampling frame of 278 clinicians was drawn from professional networks and academic institutions, covering a cross-section of these specialties. The required sample size was calculated using the standard formula for a finite population:

$$n=\frac{Z^{2}\times p\times q\times N}{e^{2}\left(N-1\right)+Z^{2}\times p\times q}$$

Where:

•
n = required sample size
•
N = total population of orthodontists and oral and maxillofacial surgeons in Saudi Arabia (≈ 1,400)
•
Z = 1.96 (for 95% confidence level)
•
p = 0.5 (assumed proportion)
•
q=1−p

•
e = 0.05 (margin of error)

Based on these parameters, the minimum required sample size was *n* = 302. Although the theoretical minimum sample size was calculated as 302 participants, the final number of complete responses obtained was lower due to the voluntary nature of survey participation and the specialized target population. Consequently, the present study should be interpreted as an exploratory survey investigation rather than a fully powered inferential study. The statistical analyses were therefore conducted primarily to identify preliminary patterns and associations rather than to support definitive population-level conclusions. To maximize recruitment and account for expected non-response, the questionnaire was distributed to 278 clinicians who were accessible through professional networks, academic contacts, and professional organizations such as the Saudi Orthodontic Society and regional oral and maxillofacial surgery networks.

A non-probability convenience sampling method was used, which is appropriate for exploratory research involving geographically dispersed professional populations. Given the niche focus on surgical orthodontics and CAT, targeted sampling via professional channels was necessary to reach experienced clinicians. While convenience sampling may introduce selection bias, it remains an accepted approach in preliminary, perception-based clinical surveys. This approach may introduce **self-selection bias**, as clinicians with prior interest in or experience with clear aligner therapy may have been more likely to respond.

Certain inclusion criteria were set for the selection of the study population: orthodontists and oral and maxillofacial surgeons practicing in Saudi Arabia, regardless of gender, with or without previous experience in the use of clear aligners for orthognathic surgeries. Likewise, exclusion criteria were also set: practitioners unwilling to participate in the study, incomplete responses, and general dentists. A visual flowchart of the recruitment and inclusion/exclusion process is provided in Figure 1, illustrating the total number of professionals contacted, eligibility screening, and final response count. Given the achieved sample size, the results of inferential statistical analyses presented in this study should be interpreted as exploratory and hypothesis-generating rather than confirmatory.

**Figure 1.**
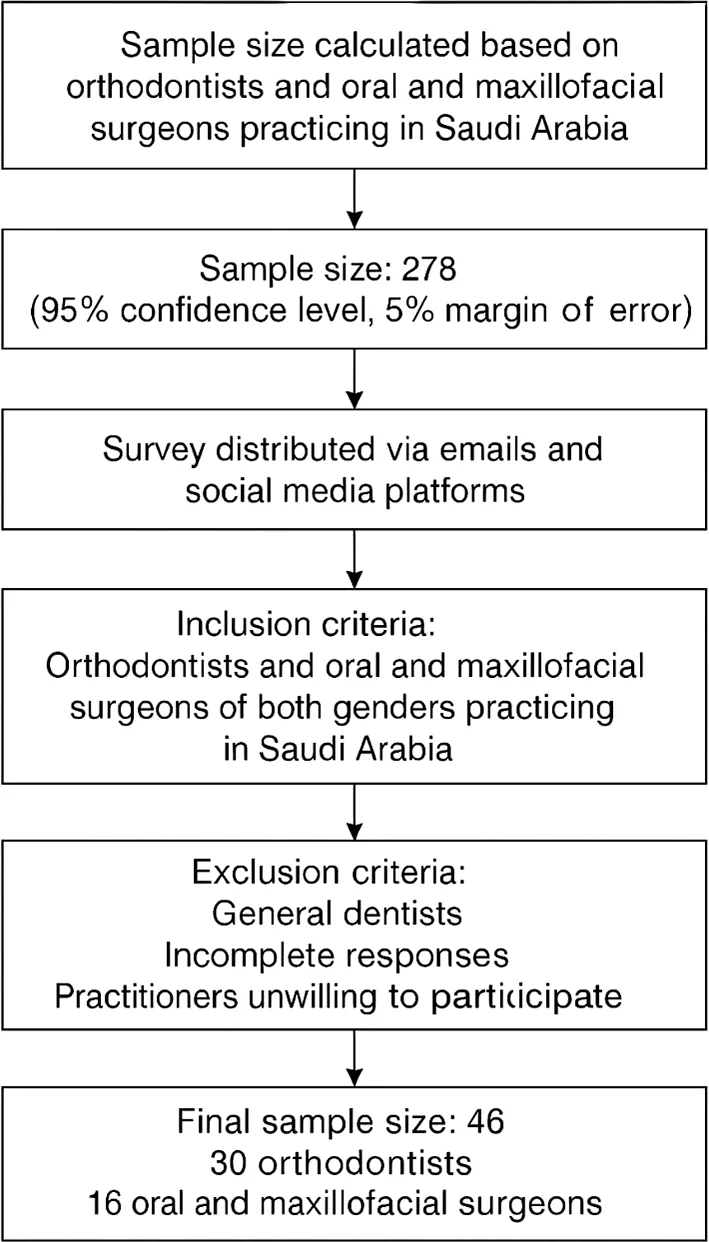
Flowchart of participant recruitment and selection process.

This figure illustrates the sampling method, survey distribution, inclusion and exclusion criteria, and the final sample of orthodontists and oral and maxillofacial surgeons who participated in the study.

Given the 16.5% response rate, potential non-response and self-selection biases were acknowledged. Low participation rates are common in clinician-based surveys due to professional workload and voluntary participation. To reduce bias, invitations were distributed through multiple channels (academic institutions, the Saudi Orthodontic Society, and social media), and anonymity was maintained to encourage honest responses. Non-parametric tests were applied to accommodate small-sample variability, and results were interpreted descriptively within an exploratory framework.

Although only 46 valid responses were obtained compared to the calculated minimum of 302, this reflects the limited pool of eligible specialists in Saudi Arabia. The small sample size reduces statistical power and increases the risk of Type II error; however, the study was designed as exploratory rather than inferential. Despite these limitations, the findings provide valuable pilot data on clinician awareness and readiness to integrate Clear Aligner Therapy (CAT) in orthognathic practice and establish a foundation for future multicenter research with adequate power and broader participation.

### Data collection instrument

The questionnaire comprised four sections. The first section includes demographic data. The second section was related to the current experience in orthognathic surgery and CAT. The third section addressed the knowledge of CAT used in orthognathic surgery patients. The fourth section was related to CAT practice in orthognathic surgery patients. To account for differences in expertise, the questionnaire included items assessing each respondent’s level of clinical involvement in orthognathic surgery cases such as the number of surgical cases managed per year, their role in interdisciplinary treatment planning, and prior use of CAT in such cases. Participants were also asked about their familiarity with digital and surgery-first workflows. These measures helped ensure that responses reflected informed perspectives and allowed subgroup comparisons based on clinical experience.

Because the survey was designed to obtain an initial overview of clinician experience and perceptions, some orthognathic treatment scenarios involving CAT, including surgery-first approaches, hybrid workflows, adjunctive TAD-supported mechanics, and combined fixed appliance protocols, were captured within broader survey categories rather than analyzed as fully separate protocol-specific pathways.

### Validity and reliability

The content validity of the questionnaire was assessed by two subject-matter experts an orthodontist and an oral and maxillofacial surgeon who evaluated each item for clarity, relevance, and alignment with the study objectives. Based on their feedback, minor linguistic modifications were made, and two items were reworded to eliminate ambiguity, particularly regarding post-surgical treatment steps. No items were removed, as all were deemed essential to the study scope. To assess reliability, the questionnaire was pilot-tested with 20 participants, who completed the survey twice, one week apart. Responses were compared using Cohen’s Kappa statistic, which demonstrated substantial agreement (κ > 0.70), confirming good test–retest reliability of the instrument.

### Statistical analysis

Data were analyzed using IBM SPSS Statistics version 27. Descriptive statistics were used as the primary analytical approach, with categorical variables summarized as frequencies and percentages and continuous or ordinal summary measures presented as appropriate. Because the achieved sample size was substantially smaller than the planned sample and several response categories contained low frequencies, all inferential analyses were treated as exploratory and interpreted with caution.

Where applied, chi-square or Fisher’s exact tests were used only for exploratory comparisons between selected categorical variables, depending on cell distribution and expected frequencies. Any p-values reported should therefore be interpreted as exploratory rather than confirmatory. A univariate exploratory regression model was also conducted to examine whether selected participant characteristics were associated with CAT usage. Given the limited sample size, possible sparse data structure, and the exploratory nature of the study, regression findings were not intended to establish predictive relationships but rather to identify preliminary patterns that may inform future research.

The CAT usage score used in the exploratory regression was derived from questionnaire responses reflecting reported experience with CAT in orthognathic surgery. This score was created for the purposes of the present survey and should not be interpreted as a previously validated scale. Accordingly, all model estimates, including R ^2^ values, should be interpreted with substantial caution.

For these exploratory categorical analyses, the null hypothesis was that no association existed between the variables being compared. These comparisons were included only where they were considered clinically relevant to the study objective of describing patterns of CAT use in orthognathic practice.

## Results

A total of 46 complete responses were obtained from 278 invitations, yielding a response rate of 16.5%. Given the limited sample size, the following results are presented primarily as descriptive observations. While this response rate is modest, it is within the range reported for similar surveys targeting specialized medical professionals. The study included 46 participants, predominantly male (67.4%) and aged mostly between 31-40 years (54.3%). Most participants had 6-15 years of professional experience (58.7%) and were mainly orthodontists (65.2%) or oral and maxillofacial surgeons (34.8%) (Supplementary Table S1). Among orthodontists (N = 30), 66.7% saw 1-4 orthognathic surgery patients monthly, and 56.7% practiced clear aligner therapy (CAT) with 1-10 patients weekly. However, 80% of respondents reported no prior experience using CAT in orthognathic surgery patients, indicating limited exposure to this treatment modality. (Supplementary Table S2). These findings should be interpreted within the context of the limited sample size, which may affect the reliability and external validity of the results. Detailed demographic and baseline characteristics are provided in **Supplementary Tables S1 and S2**.

Among oral surgeons (N = 16), half examined 5-10 orthognathic surgery patients weekly. About 44% treated 1-4 patients with CAT, while another 44% reported no CAT use, suggesting variable levels of clinical exposure to CAT among surgeons ( Table 1).

**Table 1.** The current experience of the oral and maxillofacial surgeons in orthognathic surgery and use of Clear Aligner Therapy (CAT) (N = 16).

Categories	Frequency range (No. of patients)	Frequency (n)	Percentage (%)	p-value
How often do you see orthognathic surgery patients in your practice weekly?	1-4	5	31.2	0.30 CI: (25.5, 74.5)
	5-10	8	50	
	>15	3	18.8	
How many orthognathic surgery patients have you treated using clear aligners (pre or post-surgery)	1-4	7	43.8	0.02 CI: (19.5, 68.1)
	5-10	1	6.2	
	>15	1	6.2	
	None	7	43.8	

Statistical test: Chi-square test; Abbreviations: CI, Confidence Interval; p, probability value.

Orthodontists expressed challenges primarily in pre-surgical alignment, leveling, and space closure, with 26.7%-36.7% agreeing on difficulties. Most agreed that malocclusion type influences the choice between CAT and fixed appliances (83.3%) ( Table 2).

**Table 2.** Knowledge of the orthodontists regarding CAT (N = 30).

Questions	Response (frequency (%))						
	Agree	Strongly agree	Disagree	Strongly disagree	Neutral	I don’t know	Not applicable
It is challenging to achieve the needed pre-surgical alignment required for accurate surgical setup	5 (16.7)	5 (16.7)	8 (26.7)	2 (6.7)	6 (20)	3 (10)	1 (3.3)
It is challenging to achieve the needed pre-surgical levelling to facilitate orthognathic surgical movements	7 (23.3)	1 (3.3)	7 (23.3)	2 (6.7)	8 (26.7)	4 (13.3)	1 (3.3)
It is challenging to achieve the needed pre surgical space closure necessary for orthognathic preparation	6 (20)	5 (16.7)	10 (33.3)	1 (3.3)	4 (13.3)	3 (10)	1 (3.3)
The type of malocclusion plays a role in the treatment choice (CAT vs fixed appliance) for surgical planning	8 (26.7)	17 (56.7)	0 (0)	1 (3.3)	2 (6.7)	1 (3.3)	1 (3.3)
CAT takes longer pre-surgical time compared with fixed appliances	4 (13.3)	3 (10)	8 (26.7)	1 (3.3)	11 (36.7)	1 (3.3)	2 (6.7)
CAT pre-surgical outcomes are similar to fixed appliance treatment	5 (16.7)	4 (13.3)	5 (16.7)	3 (10)	7 (23.3)	2 (6.7)	4 (13.3)
It is challenging to achieve the needed post-surgical refinement using CAT	5 (16.7)	3 (10)	7 (23.3)	2 (6.7)	7 (23.3)	4 (13.3)	2 (6.7)
It is challenging to obtain post-surgical occlusion and stability with CAT	10 (33.3)	3 (10)	8 (26.7)	1 (3.3)	3 (10)	3 (10)	2 (6.7)

Statistical test: Chi-square test; Abbreviations: CAT, Clear Aligner Therapy; p, probability value.

Oral surgeons highlighted challenges in surgical planning and intraoperative fixation, but many disagreed that final stable occlusion post-surgery was difficult to achieve ( Table 3).

**Table 3.** Knowledge of the oral and maxillofacial surgeons regarding CAT (N = 16).

Questions	Response (frequency (%))					
	Agree	Strongly agree	Disagree	Strongly disagree	Neutral	I don’t know
It is challenging to perform the surgical planning in CAT	4 (25)	1 (6.2)	2 (12.5)	3 (18.8)	5 (31.2)	1 (6.2)
It is challenging to achieve intraoperative intermaxillary fixation (IMF) in CAT	5 (31.2)	3 (18.8)	3 (18.8)	2 (12.5)	2 (12.5)	1 (6.2)
It is challenging to use the interim splint during the surgical procedure in CAT	3 (18.8)	2 (12.5)	2 (12.5)	3 (18.8)	5 (31.2)	1 (6.2)
It is challenging to obtain a final stable occlusion after the surgery	3 (18.8)	2 (12.5)	6 (37.5)	1 (6.2)	4 (25)	0 (0)
CAT can be used with segmental osteotomies procedures	5 (31.2)	3 (18.8)	3 (18.8)	3 (18.8)	1 (6.2)	1 (6.2)
CAT can be used with excessive maxillary or mandibular movements (advancement and setback)	6 (37.5)	4 (25)	1 (6.2)	3 (18.8)	1 (6.2)	1 (6.2)
CAT can be used with excessive rotational movements	7 (43.8)	1 (6.2)	2 (12.5)	2 (12.5)	3 (18.8)	1 (6.2)
CAT can be used in surgery first concept	7 (43.8)	3 (18.8)	1 (6.2)	1 (6.2)	3 (18.8)	1 (6.2)
CAT can be used in mandible first concept	6 (37.5)	2 (12.5)	3 (18.8)	1 (6.2)	3 (18.8)	1 (6.2)

Statistical test: Chi-square test; Abbreviations: CAT, Clear Aligner Therapy; IMF, Intermaxillary Fixation; CI, Confidence Interval; p, probability value.

No significant difference in overall knowledge of CAT between orthodontists and oral surgeons was found (p = 0.59), suggesting comparable expertise levels ( Table 4).

**Table 4.** Comparison of average knowledge scores between orthodontists and oral surgeons (Mann-Whitney U test).

Question item	Orthodontists (Mean score) CI: (3.42, 4.26)	Oral surgeons (Mean score) CI: (3.95, 4.48)	p-value
Q1	3.72	3.56	0.59 (3.42, 4.26)
Q2	3.48	4.25	
Q3	3.97	4.44	
Q4	4.86	3.94	
Q5	3.41	4.31	
Q6	3.65	4.5	
Q7	3.39	4.25	
Q8	4.25	4.44	
Q9		4.25	

Statistical test: Mann–Whitney U test; Abbreviations: CAT, Clear Aligner Therapy; CI, Confidence Interval; p, probability value.

Post-surgical management commonly required fixed appliances and temporary anchorage devices (TADs), but only 6.7% felt fully confident in using CAT in orthognathic cases ( Table 5).

**Table 5.** Responses of study orthodontists CAT practice in orthognathic surgery patients (N = 30).

Question	Responses (frequency (%))						
	Always	Often	Sometimes	Never	Rarely	I don’t know	Not Applicable
The case complexity drive your decision in selecting CAT as a treatment choice	18 (60)	4 (13.3)	4 (13.3)	1 (3.3)	1 (3.3)	1 (3.3)	1 (3.3)
The availability of the needed digital tools affect your decision in selecting CAT as a treatment of choice	8 (26.7)	6 (20)	9 (30)	3 (10)	2 (6.7)	1 (3.3)	1 (3.3)
The cost of the treatment plays a major role in selecting CAT as a treatment choice	10 (33.3)	7 (23.3)	9 (30)	2 (6.7)	0 (0)	1 (3.3)	1 (3.3)
The patient demands drive your decision in selecting CAT as a treatment choice	8 (26.7)	10 (33.3)	8 (26.7)	0 (0)	1 (3.3)	2 (6.7)	1 (3.3)
The skills of the maxillofacial surgeon drive your decision in selecting CAT as a treatment choice	5 (16.7)	8 (26.7)	8 (26.7)	4 (13.3)	2 (6.7)	1 (3.3)	1 (3.3)
The cooperation of the maxillofacial surgeon drive your decision in selecting CAT as a treatment choice	9 (30)	7 (23.3)	8 (26.7)	2 (6.7)	1 (3.3)	1 (3.3)	2 (6.7)
The patient cooperation affect your decision in selecting CAT as a treatment (before or after surgery)	16 (53.3)	10 (33.3)	1 (3.3)	1 (3.3)	0 (0)	1 (3.3)	1 (3.3)
The reduced time of the treatment plays a major role in selecting CAT as a treatment choice	9 (30)	5 (16.7)	9 (30)	3 (10)	2 (6.7)	1 (3.3)	1 (3.3)
The reduced number of visits of the treatment plays a major role in selecting CAT as a treatment choice	8 (26.7)	5 (16.7)	10 (33.3)	3 (10)	2 (6.7)	1 (3.3)	1 (3.3)
The reduced pain during treatment plays a major role in selecting CAT as a treatment choice	4 (13.3)	3 (10)	9 (30)	6 (20)	5 (16.7)	1 (3.3)	2 (6.7)
CAT combined with fixed appliances is required before the surgery	5 (16.7)	4 (13.3)	14 (46.7)	2 (6.7)	2 (6.7)	2 (6.7)	1 (3.3)
Fixed appliance bonding is needed for post-surgical management	4 (13.3)	7 (23.3)	16 (53.3)	0 (0)	1 (3.3)	1 (3.3)	1 (3.3)
Temporary anchorage device (TADS) are needed for post-surgical management	0 (0)	5 (16.7)	18 (60)	0 (0)	5 (16.7)	1 (3.3)	1 (3.3)
You are confident of using CAT in orthognathic patients	2 (6.7)	4 (13.3)	13 (43.3)	3 (10)	3 (10)	4 (13.3)	1 (3.3)

Statistical test: Chi-square test; Abbreviations: CAT, Clear Aligner Therapy; TADs, Temporary Anchorage Devices; p, probability value.

Fixed appliances were often requested pre-surgery (43.8%), and intraoperative fixation relied mostly on IMF screws (37.5%) ( Table 6).

**Table 6.** Responses of study oral and maxillofacial surgeons CAT practice in orthognathic surgery patients (N = 16).

Question	Responses (frequency (%))						
	Always	Often	Sometimes	Never	Rarely	I don’t know	Not applicable
The surgery time is increased in CAT cases	1 (6.2)	2 (12.5)	3 (18.8)	3 (18.8)	5 (31.2)	2 (12.5)	0 (0)
It is difficult to control the post-operative occlusion on CAT patients	2 (12.5)	2 (12.5)	8 (50)	1 (6.2)	1 (6.2)	2 (12.5)	0 (0)
How frequent you ask the orthodontist to bond fixed appliances before the surgery (if the pre-surgical orthodpntics was using clear aligner)	7 (43.8)	5 (31.2)	1 (6.2)	1 (6.2)	0 (0)	**1 (6.2)**	**1 (6.2)**
How frequent do you rely on IMF screws for intraoperative IMF	6 (37.5)	3 (18.8)	3 (18.8)	1 (6.2)	3 (18.8)	0 (0)	0 (0)
How frequent do you rely on TADS intraoperative IM	2 (12.5)	2 (12.5)	1 (6.2)	5 (31.2)	5 (31.2)	1 (6.2)	0 (0)
How frequent do you rely on archbars intraoperative IMF	1 (6.2)	3 (18.8)	2 (12.5)	5 (31.2)	4 (25)	1 (6.2)	0 (0)
The post-operative occlusion for CAT cases is guided using elastics	2 (12.5)	3 (18.8)	4 (25)	1 (6.2)	4 (25)	1 (6.2)	1 (6.2)
The post-operative occlusion for CAT is guided using wires	1 (6.2)	1 (6.2)	3 (18.8)	4 (25)	3 (18.8)	3 (18.8)	1 (6.2)
How frequent do you rely on TADS to manage post-operative occlusion relapse	1 (6.2)	3 (18.8)	4 (25)	5 (31.2)	0 (0)	2 (12.5)	1 (6.2)
How frequent do you rely on arch bars to manage post-operative relapse	2 (12.5)	1 (6.2)	2 (12.5)	7 (43.8)	2 (12.5)	1 (6.2)	1 (6.2)
How frequent do you rely on orthodontic fixed appliance to manage post-operative relapse	6 (37.5)	1 (6.2)	5 (31.2)	2 (12.5)	2 (12.5)	0 (0)	0 (0)

Statistical test: Chi-square test; Abbreviations: CAT, Clear Aligner Therapy; IMF, Intermaxillary Fixation; TADs, Temporary Anchorage Devices; p, probability value.

Age showed a statistically significant association with CAT usage among both orthodontists (p = 0.01, adjusted R ^2^ = 0.284) and oral surgeons (p = 0.02, adjusted R ^2^ = 0.25); however, given the limited sample size and exploratory design, this finding should be interpreted as hypothesis-generating rather than confirmatory. Exploratory univariate regression suggested a possible association between age and CAT usage in both professional groups; however, these findings should be interpreted with substantial caution given the small sample size and the survey-derived nature of the CAT usage variable. Gender and years of experience showed no significant correlation ( Table 7).

**Table 7.** Univariate linear regression model for correlation between CAT usage and demographical parameters.

Variable	*R ^2^*	*Adjusted R ^2^*	P-value
**Orthodontists**			
Gender	0.021	-0.014	0.44
Age	0.383	0.284	0.01
Experience	0.154	0.019	0.361
**Oral and maxillofacial surgeons**			
Gender	0.05	-0.01	0.38
Age	0.30	0.25	0.02
Experience	0.13	0.07	0.15

Statistical test: Univariate linear regression analysis; Abbreviations: CAT, Clear Aligner Therapy; R ^2^, Coefficient of Determination; p, probability value.

Exploratory univariate regression models were conducted separately for orthodontists and oral and maxillofacial surgeons using a survey-derived CAT usage variable. Given the limited sample size, these results should be interpreted cautiously and regarded as hypothesis-generating only.

## Discussion

### Overview of findings

This exploratory survey provides preliminary data on clinician knowledge, experience, and attitudes regarding the use of clear aligner therapy (CAT) in orthognathic surgery among orthodontists and oral and maxillofacial surgeons in Saudi Arabia. Although respondents showed general awareness of CAT and its emerging role in surgical orthodontics, direct clinical use in orthognathic cases remained limited. The findings should be interpreted cautiously because the achieved sample size was small, the response rate was low, and the study was not powered to support strong inferential conclusions.

Among the 278 professionals invited, 46 completed the survey, yielding a response rate of 16.5%. Most respondents reported routine exposure to orthognathic surgery patients, but many had limited or no experience using CAT in pre- or post-surgical phases. The Mann–Whitney U test did not detect a statistically significant difference in overall CAT knowledge between orthodontists and oral and maxillofacial surgeons. However, this comparison should be considered exploratory and hypothesis-generating rather than conclusive.

### Knowledge and attitudes toward CAT

The findings suggest that clinicians recognize the potential of CAT in orthognathic care but remain cautious regarding its predictability in complex surgical cases. Orthodontists reported concerns related to pre-surgical alignment, leveling, space closure, post-surgical refinement, and occlusal stability. Most respondents also agreed that malocclusion type influences the choice between CAT and fixed appliances. These perceptions are consistent with broader reports that, although CAT has become increasingly effective for mild-to-moderate malocclusions, evidence supporting its routine use in surgical orthodontics remains limited and more protocol-dependent. ^20– 24^

This cautious attitude is understandable in light of the biomechanical demands of orthognathic preparation. Existing literature continues to identify limitations of aligners in root control, torque expression, vertical correction, and management of complex three-dimensional movements, especially when precise decompensation and stable post-surgical occlusion are required. ^25^ The lack of significant knowledge differences between orthodontists and surgeons in the present study may also reflect the increasingly interdisciplinary nature of digital planning and simulation in orthognathic workflows. ^26– 28^

### Clinical challenges and workflow uncertainties

The present findings indicate that CAT integration into orthognathic surgery remains clinically and logistically challenging. Respondents expressed uncertainty regarding whether CAT prolongs pre-surgical treatment, and many reported concerns about pre-surgical setup, intraoperative fixation, and post-operative occlusal control. These concerns are consistent with prior reports describing CAT in orthognathic practice as promising but technically demanding, particularly when compared with conventional fixed appliance workflows that have a longer history and wider biomechanical versatility. ^7, 10, 11, 29^

Several respondents also indicated that hybrid approaches may be necessary in selected cases. The continued need for fixed appliances, temporary anchorage devices, IMF screws, elastics, or arch bars in perioperative management suggests that CAT is often being viewed not as a complete substitute for fixed appliances, but rather as part of a blended or case-specific treatment strategy. This interpretation aligns with emerging reports from surgery-first and digitally assisted orthognathic protocols, where aligners may be combined with adjunctive mechanics, splint modifications, or TAD-supported approaches to improve predictability. ^26– 28^

Our findings appear to align with international observations suggesting that CAT may represent a promising but technically challenging modality that demands interdisciplinary teamwork, strong digital infrastructure and flexible case-by-case planning.

### Practice preferences and treatment decision factors

The study further suggests that treatment selection is influenced by case complexity, patient demand, comfort, cost, cooperation, and digital workflow access. This is consistent with international survey data showing that clinicians often reserve CAT for cases in which aesthetics, convenience, and oral hygiene are prioritized, while remaining more cautious in severe skeletal discrepancies or situations requiring highly precise post-surgical occlusal control. ^30, 31^ Although patient demand for less visible appliances continues to grow, especially among adults, clinician confidence in CAT for orthognathic cases appears to lag behind its wider uptake in routine orthodontic practice. This gap likely reflects not only technical limitations, but also the continuing absence of universally accepted protocols for case selection, sequencing, fixation, and refinement in surgical CAT workflows. ^22– 28, 32^

It is important to note that the convenience sampling strategy and relatively small number of respondents may limit the generalizability of these findings to the broader population of orthodontists and oral and maxillofacial surgeons in Saudi Arabia. Future studies with larger, multicenter samples are required to confirm these observations.

### Demographic trends and exploratory regression findings

Exploratory regression suggested a possible association between clinician age and CAT usage. However, this finding should not be interpreted as evidence of a stable explanatory relationship. The regression analysis was conducted on a small sample, and the CAT usage measure was survey-derived rather than externally validated. Therefore, the model may be vulnerable to instability and overfitting. The observed association is best viewed as a preliminary signal that may help generate hypotheses for future larger studies rather than as a robust determinant of CAT adoption. Similar caution is warranted when interpreting the lack of association with gender and years of experience. ^20, 33, 34^

### Implications for clinical protocols and training

The findings highlight the need for clearer clinical guidance and more structured training for the use of CAT in orthognathic surgery. For orthodontists, training should include digital workflow planning, aligner biomechanics, case selection, recognition of CAT limitations in surgical decompensation, and the appropriate use of hybrid strategies when aligners alone are insufficient. For oral and maxillofacial surgeons, training should focus more specifically on surgical coordination issues, including splint adaptation, intermaxillary fixation, post-operative elastic guidance, and management of occlusal stability in CAT-based cases. ^25, 27, 28^

Interdisciplinary collaboration remains essential because CAT-based surgical workflows require close coordination between orthodontic planning and surgical execution. Future studies should distinguish more clearly between orthodontics-first protocols, surgery-first approaches, hybrid workflows, adjunctive TAD-supported mechanics, and combinations with fixed appliances. Protocol-specific multicenter investigations with adequate statistical power are needed to determine in which clinical scenarios CAT can be applied predictably and safely in orthognathic care. ^26– 28^

Overall, the findings of the present study should be considered preliminary and primarily useful for identifying areas requiring further investigation. They do not provide definitive evidence regarding the role of CAT in orthognathic surgery, but they do offer useful early regional insight into current clinician perceptions, practice gaps, and priorities for future research. ^22– 28, 35^

### Limitations

Despite providing useful preliminary insight into clinician perspectives on clear aligner therapy (CAT) in orthognathic surgery, this study has several important limitations.

First, the achieved sample size was small. Although invitations were sent to 278 eligible clinicians, only 46 complete responses were obtained, yielding a response rate of 16.5%. While this participation rate is comparable to some clinician-based surveys, it remained substantially lower than the calculated minimum required sample size and therefore limited the statistical power, precision, and external validity of the study. As a result, the findings cannot be generalized confidently to the broader population of orthodontists and oral and maxillofacial surgeons in Saudi Arabia.

Second, the low participation rate raises the possibility of non-response and self-selection bias. Clinicians with greater familiarity, interest, or confidence in CAT may have been more likely to participate. Because the survey was anonymous, a formal non-responder analysis was not possible.

Third, the statistical analyses should be interpreted strictly as exploratory. Given the small sample size and sparse responses across some categories, inferential analyses, including chi-square testing and univariate regression, may be vulnerable to limited stability and reduced interpretability. The observed regression findings, including the reported R ^2^ value, should therefore be regarded as hypothesis-generating rather than confirmatory. Similarly, the CAT usage variable used in the exploratory regression was survey-derived and was not based on a previously validated scale, which further limits confidence in model-based interpretation.

Fourth, the study used a non-probability convenience sampling approach. Although pragmatic and commonly used in exploratory survey research, this method limits sample representativeness and further constrains generalizability. The findings should therefore be viewed as preliminary rather than representative of national practice patterns.

Fifth, the questionnaire captured clinician perspectives on CAT use in orthognathic surgery broadly, but it did not fully distinguish between all relevant treatment scenarios, such as surgery-first approaches, hybrid workflows, adjunctive TAD-supported mechanics, and combinations with fixed appliances. Grouping these scenarios within broader survey categories may have reduced the conceptual precision of the findings and limited protocol-specific interpretation.

Sixth, the study relied on self-reported responses, which may be influenced by recall bias, subjective interpretation, and possible overestimation or underestimation of familiarity and competence with CAT-related workflows.

Finally, although international literature was used to contextualize the findings, such comparisons should be interpreted cautiously because differences in aligner systems, digital infrastructure, training exposure, and regulatory or economic environments may influence adoption patterns and workflow feasibility across countries.

Taken together, these limitations indicate that the present findings should be interpreted as preliminary, descriptive, and hypothesis-generating rather than definitive. Nevertheless, the study contributes useful early regional data and identifies important educational, clinical, and research gaps that may inform future multicenter studies with stronger methodological and statistical power.

## Conclusion

Within the limitations of this exploratory survey, clinicians in Saudi Arabia appeared to report limited direct experience with the use of clear aligner therapy in orthognathic surgery, despite increasing interest in digital orthodontic-surgical workflows. The findings should be interpreted cautiously because of the small achieved sample size, low response rate, and limited inferential robustness. Rather than providing definitive evidence, this study offers preliminary insight that may help guide future multicenter, adequately powered, protocol-specific investigations into the role of CAT in orthognathic surgery.

## Underlying data

**Repository name:** The Use of Clear Aligner Therapy for Orthognathic Surgery Patients: A Cross-Sectional Survey Among Saudi Orthodontics and Oral and Maxillofacial Surgeons. https://doi.org/10.5281/zenodo.16977338

The project contains the following underlying data:

FINAL The use of clear aligner therapy for orthognathic surgery patients (Responses)-3.xlsx (raw).

Supplementary Tables.docx **(Tables).**

Data are available under the terms of the Creative Commons Zero “No rights reserved” data waiver (CC0 1.0 Public domain dedication).

## Software availability

The statistical analysis for this study was performed using the Statistical Package for the Social Sciences (SPSS), version 27 (SPSS Inc., Chicago, IL, USA). The software used for digital treatment planning and data collection, including the Google Forms platform for survey distribution, are freely available for public use. No specialized or proprietary software was required for the completion of this study beyond those mentioned above.

## Supplementary materials

The complete survey questionnaire used in this study is provided in *Supplementary Table S1.* It includes all sections used to assess demographics, clinical experience, knowledge, and attitudes toward the use of clear aligner therapy (CAT) in orthognathic surgery. An anonymized dataset of all participant responses (n = 46), excluding any personal identifiers, is provided as *Supplementary Table S2* to support transparency and allow for further secondary analysis.
