# Supplementary material for: The Use of Clear Aligner Therapy for Orthognathic Surgery Patients: A Cross-Sectional Survey Among Saudi Orthodontists and Oral and Maxillofacial Surgeons
Source: F1000Res. 2026 Oct 14;14:1001. Originally published 2025 Sep 29. [Version 5] doi: 10.12688/f1000research.170095.5 (PMC13687734; doi:10.12688/f1000research.170095.5)
Supplement: Peer review history

# Reviewer report: Reviewer response for version 5

**DOI:** 10.5256/f1000research.201555.r530022

**Published:** 2026-10-22

**Authors:** Tiago Fialho

**Affiliations:** 1 Department of Orthodontics, Bauru Dental School, São Paulo University, Bauru, SP, Brazil

*Thank you for the opportunity to review Version 5 of this manuscript. I have carefully assessed the revised manuscript in light of the concerns raised in my previous report and the authors’ responses.*

**

*The authors have made substantial and appropriate revisions. The study is now consistently framed as an exploratory cross-sectional survey, and the limitations related to the small achieved sample size, low response rate, convenience sampling, self-selection bias, and limited inferential robustness are explicitly acknowledged throughout the Abstract, Methods, Results, Discussion, Limitations, and Conclusion. The regression analysis and the survey-derived CAT usage score are now described with appropriate caution, and the interpretation of the age-related association has been reframed as hypothesis-generating rather than definitive. The Discussion and Limitations sections have also been streamlined and are more focused on the actual findings.*

**

*The manuscript does not provide definitive evidence on the use of clear aligner therapy in orthognathic surgery, and it does not claim to do so. Within the scope of an exploratory survey, however, it offers useful preliminary regional data and identifies relevant clinical, educational, and research gaps. The open availability of the dataset and survey instrument further supports transparency.*

**

***Residual comments — non-essential***

1. *Tables 1–6 still report chi-square p-values in the context of small cell frequencies. Although the exploratory framing and explicit limitations mitigate this concern, future reports would benefit from Fisher’s exact tests or predominantly descriptive reporting.*
2. *The Methods refer to “non-parametric tests,” while several tables report chi-square analyses. This wording could be harmonized for consistency.*
3. *The regression findings should remain clearly labelled as exploratory and hypothesis-generating; the current version already does this appropriately.*
4. *The broad grouping of different CAT-related orthognathic protocols is now acknowledged as a limitation. Future protocol-specific studies would improve interpretability.*

*These points do not require further essential revision and may be addressed at the editorial/proof stage or in future work.*

Is the work clearly and accurately presented and does it cite the current literature?

Partly

If applicable, is the statistical analysis and its interpretation appropriate?

Partly

Are all the source data underlying the results available to ensure full reproducibility?

Yes

Is the study design appropriate and is the work technically sound?

Partly

Are the conclusions drawn adequately supported by the results?

Partly

Are sufficient details of methods and analysis provided to allow replication by others?

Partly

Reviewer Expertise:

NA

I confirm that I have read this submission and believe that I have an appropriate level of expertise to confirm that it is of an acceptable scientific standard.

# Reviewer report: Reviewer response for version 5

**DOI:** 10.5256/f1000research.201555.r530523

**Published:** 2026-10-21

**Authors:** Arya Brahmanta

**Affiliations:** 1 Universitas Hang Tuah, Surabaya, East Java, Indonesia

This manuscript investigates the knowledge, clinical experience, attitudes, and practices of orthodontists and oral and maxillofacial surgeons in Saudi Arabia regarding the application of clear aligner therapy (CAT) in orthognathic surgery patients.In my assessment, Version 5 is sufficiently clear, transparent, and scientifically acceptable as an exploratory cross-sectional survey. The conclusions are appropriately limited to the evidence presented, and the manuscript makes a useful contribution to understanding clinician perspectives on clear aligner therapy in orthognathic surgery.No further essential revisions are requested.

Is the work clearly and accurately presented and does it cite the current literature?

Yes

If applicable, is the statistical analysis and its interpretation appropriate?

Yes

Are all the source data underlying the results available to ensure full reproducibility?

Yes

Is the study design appropriate and is the work technically sound?

Yes

Are the conclusions drawn adequately supported by the results?

Yes

Are sufficient details of methods and analysis provided to allow replication by others?

Yes

Reviewer Expertise:

Orthodontic

I confirm that I have read this submission and believe that I have an appropriate level of expertise to confirm that it is of an acceptable scientific standard.

# Reviewer report: Reviewer response for version 4

**DOI:** 10.5256/f1000research.200348.r482398

**Published:** 2026-05-09

**Authors:** Abdelrahman MA Mohamed

**Affiliations:** 1 Orthodontics, Royal College of Surgeons of Edinburgh, Edinburgh, UK

I would like to thank the editor very much for giving me the opportunity to review this paper. 

**1. Results: **Tables s1 and s2 definitions in the footprint need to be added ( P value, CI).

 2. Discussion and Limitations sections are overly long and contains repeated information under different subheadings, leading to unnecessary redundancy.

 The manuscript is generally acceptable after revision; however, an additional reviewer opinion may help strengthen the editorial assessment, particularly regarding the sample size, response rate, and the organization and redundancy within the Discussion section.

Is the work clearly and accurately presented and does it cite the current literature?

Partly

If applicable, is the statistical analysis and its interpretation appropriate?

Partly

Are all the source data underlying the results available to ensure full reproducibility?

No

Is the study design appropriate and is the work technically sound?

Partly

Are the conclusions drawn adequately supported by the results?

No

Are sufficient details of methods and analysis provided to allow replication by others?

Partly

Reviewer Expertise:

Orthodontics.

I confirm that I have read this submission and believe that I have an appropriate level of expertise to confirm that it is of an acceptable scientific standard.

# Response

**Published:** 2026-05-13

**Authors:** Ahmad Assari

**Affiliations:** Not Applicable, Saudi Arabia

**Response to Reviewer**

 We sincerely thank the reviewer for the careful evaluation of our manuscript and for the constructive suggestions. We also appreciate the overall positive assessment that the manuscript is generally acceptable after revision. We have carefully addressed each point raised, as detailed below.

**1. Results: Tables S1 and S2 definitions in the footnote need to be added (P value, CI).**

**Response:**

 We agree and thank the reviewer for identifying this issue. In the revised version, we added clear footnote definitions for the statistical abbreviations used in Supplementary Tables S1 and S2, including CI and p-value, to improve clarity and consistency of presentation.

**Changes made:**

- Added footnote definitions to Supplementary Tables S1 and S2 as follows:
  CI, confidence interval; p-value, probability value.

**2. Discussion and Limitations sections are overly long and contain repeated information under different subheadings, leading to unnecessary redundancy.**

**Response:**

 We agree with this important comment. In the revised manuscript, both the Discussion and Limitations sections were substantially streamlined to improve focus, reduce redundancy, and enhance readability. Repeated statements regarding sample size, response rate, exploratory interpretation, and generalizability were consolidated. Several discussion subsections that were more narrative or repetitive in nature were removed or merged, and the revised Discussion now focuses more directly on the study findings, their cautious interpretation, and implications for future research and training. The Limitations section was also revised so that each major limitation is stated once in a more concise and organized manner.

**Changes made:**

- Shortened and reorganized the Discussion section.
- Removed repeated or overlapping discussion content across multiple subheadings.
- Consolidated cautionary statements regarding the exploratory nature of the study.
- Streamlined the Limitations section to reduce redundancy and improve clarity.

**3. The manuscript is generally acceptable after revision; however, an additional reviewer opinion may help strengthen the editorial assessment, particularly regarding the sample size, response rate, and the organization and redundancy within the Discussion section.**

**Response:**

 We appreciate this observation. In the revised manuscript, we further emphasized that the study should be interpreted strictly as exploratory, given the small achieved sample size and low response rate. This cautious framing has been maintained consistently across the Abstract, Methods, Statistical Analysis, Results, Discussion, Limitations, and Conclusion. We also revised the organization of the Discussion to improve overall flow and reduce redundancy, while ensuring that the implications of the sample size and response rate remain clearly acknowledged.

**Changes made:**

- Further reinforced the exploratory nature of the study throughout the manuscript.
- Maintained explicit acknowledgment of the limitations related to the small achieved sample size and low response rate.
- Improved the organization and readability of the Discussion section.

We thank the reviewer again for these valuable comments, which helped improve the clarity, structure, and overall presentation of the manuscript.

# Reviewer report: Reviewer response for version 4

**DOI:** 10.5256/f1000research.200348.r482399

**Published:** 2026-05-08

**Authors:** Tiago Fialho

**Affiliations:** 1 Department of Orthodontics, Bauru Dental School, São Paulo University, Bauru, SP, Brazil

The revised manuscript demonstrates substantial improvement compared with the previous versions. The authors have clearly made significant efforts to address prior reviewer comments, particularly regarding transparency of methodology, clarification of study limitations, restructuring of the discussion, and repositioning of the study as exploratory in nature. The manuscript now presents a more balanced and scientifically cautious interpretation of the findings, which considerably strengthens its overall credibility.

 The topic remains clinically relevant and timely, particularly considering the increasing global interest in the integration of Clear Aligner Therapy (CAT) into orthognathic surgery workflows. The manuscript also contributes preliminary regional data from Saudi Arabia, where evidence on this topic remains limited.

 Nevertheless, several methodological and statistical concerns persist and should be acknowledged as important limitations of the study.

 The principal limitation remains the substantially insufficient sample size. Although the authors appropriately calculated a minimum required sample of 302 participants, only 46 complete responses were ultimately obtained. While the authors correctly reframed the study as exploratory and acknowledged the reduced statistical power, the manuscript still includes inferential analyses that may overextend the interpretability of the data. Specifically, the use of multiple chi-square analyses and univariate linear regression models appears methodologically fragile considering the small sample size and low cell frequencies observed in several tables.

 In Tables 1 through 6, p-values are repeatedly presented without a clearly defined null hypothesis or clinically meaningful comparative framework. In several instances, the statistical purpose of the chi-square analyses is unclear, particularly because many of the presented variables are descriptive in nature rather than comparative. Additionally, the small expected frequencies in multiple categories may violate assumptions required for chi-square testing. In this context, either Fisher’s exact test or purely descriptive reporting would likely have been more appropriate.

 Similarly, the regression analysis should be interpreted with substantial caution. The manuscript does not fully explain how the composite “CAT usage score” was constructed or validated, and the limited sample size raises concerns regarding model stability, overfitting, and reliability of the reported R² values. Although the authors appropriately characterize these findings as exploratory, some statements regarding explanatory power remain somewhat stronger than the available data can robustly support.

 The discussion section is considerably improved; however, it remains somewhat lengthy and occasionally extends beyond the direct findings of the study. Certain sections resemble a narrative literature review rather than a focused interpretation of the collected data. Some speculative statements regarding clinician behavior, digital workflow adoption, and regional sociocultural trends are not directly supported by the study methodology and should therefore be interpreted cautiously.

 Another conceptual limitation is the broad inclusion of different orthognathic protocols involving CAT, including surgery-first approaches, hybrid protocols, adjunctive TAD usage, and combined fixed appliance mechanics. The questionnaire appears to group these clinical scenarios together without sufficiently distinguishing between them, which may limit the precision of the conclusions.

 Despite these limitations, the authors should be commended for explicitly acknowledging most methodological weaknesses, including selection bias, low response rate, limited external validity, and the exploratory nature of the statistical analyses. The availability of open data and the transparent reporting of limitations further strengthen the integrity of the work.

 Overall, this manuscript should not be interpreted as providing definitive evidence regarding the use of CAT in orthognathic surgery. However, within the context of an exploratory survey study and considering the publication model of F1000Research, the work provides useful preliminary insights and may serve as a valuable foundation for future multicenter investigations with adequate statistical power.

 Based on the improvements introduced in Version 4 and considering the remaining limitations described above, my recommendation is:

 Approved with Reservations.

 I encourage the authors to consider additional refinement of the statistical presentation and further reduction of speculative discussion sections in future revisions or subsequent related publications.

Is the work clearly and accurately presented and does it cite the current literature?

Partly

If applicable, is the statistical analysis and its interpretation appropriate?

Partly

Are all the source data underlying the results available to ensure full reproducibility?

Yes

Is the study design appropriate and is the work technically sound?

Partly

Are the conclusions drawn adequately supported by the results?

Partly

Are sufficient details of methods and analysis provided to allow replication by others?

Partly

Reviewer Expertise:

Orthodontics; Orthodontic Aligners, Orthodontic mechanics

I confirm that I have read this submission and believe that I have an appropriate level of expertise to confirm that it is of an acceptable scientific standard, however I have significant reservations, as outlined above.

# Response

**Published:** 2026-05-13

**Authors:** Ahmad Assari

**Affiliations:** Not Applicable, Saudi Arabia

**Response to Reviewer**

 We sincerely thank the reviewer for the careful, thoughtful, and constructive evaluation of our revised manuscript. We greatly appreciate the recognition that the manuscript has improved substantially compared with the previous versions, particularly in terms of methodological transparency, clarification of study limitations, restructuring of the discussion, and clearer positioning of the work as exploratory in nature. We also appreciate the reviewer’s acknowledgement of the clinical relevance and timeliness of the topic, as well as the value of providing preliminary regional data from Saudi Arabia.

 We carefully considered all comments and revised the manuscript accordingly. Our detailed point-by-point responses are provided below.

**1. Reviewer comment**

**The revised manuscript demonstrates substantial improvement compared with the previous versions. The authors have clearly made significant efforts to address prior reviewer comments, particularly regarding transparency of methodology, clarification of study limitations, restructuring of the discussion, and repositioning of the study as exploratory in nature. The manuscript now presents a more balanced and scientifically cautious interpretation of the findings, which considerably strengthens its overall credibility.**

**Response:**

 We sincerely thank the reviewer for this encouraging assessment. We are grateful that the revisions made in response to earlier comments have improved the transparency, balance, and scientific caution of the manuscript. We have continued to refine the revised version to ensure that the study is consistently presented as exploratory and that its limitations are clearly and transparently acknowledged throughout.

****2.** **Reviewer comment****

**The topic remains clinically relevant and timely, particularly considering the increasing global interest in the integration of Clear Aligner Therapy (CAT) into orthognathic surgery workflows. The manuscript also contributes preliminary regional data from Saudi Arabia, where evidence on this topic remains limited.**

**Response:**

 We thank the reviewer for highlighting the clinical relevance and timeliness of this topic. We agree that the evolving role of CAT in orthognathic workflows warrants investigation, particularly in regions where evidence remains limited. We have retained this contextual relevance in the Introduction and Discussion while ensuring that our findings are framed appropriately as preliminary regional observations rather than definitive conclusions.

**3. Reviewer comment**

**Nevertheless, several methodological and statistical concerns persist and should be acknowledged as important limitations of the study.**

**Response:**

 We agree. In the revised manuscript, we have further strengthened the acknowledgment of methodological and statistical limitations. These are now stated more explicitly in the Methods, Statistical Analysis, Discussion, and Limitations sections, including the low achieved sample size, limited statistical power, exploratory nature of inferential testing, potential selection bias, and limited external validity.

**4. Reviewer comment**

 The principal limitation remains the substantially insufficient sample size. Although the authors appropriately calculated a minimum required sample of 302 participants, only 46 complete responses were ultimately obtained. While the authors correctly reframed the study as exploratory and acknowledged the reduced statistical power, the manuscript still includes inferential analyses that may overextend the interpretability of the data. Specifically, the use of multiple chi-square analyses and univariate linear regression models appears methodologically fragile considering the small sample size and low cell frequencies observed in several tables.

**Response:**

 We fully agree. In the revised manuscript, we have further clarified that the final sample size was substantially below the calculated target and that this limited statistical power, precision, and inferential robustness. We now emphasize more consistently that all inferential analyses were conducted for exploratory purposes only and should not be interpreted as confirmatory. We also reduced interpretive emphasis on inferential findings throughout the manuscript.

 Changes made:

- Revised the Abstract, Discussion, Limitations, and Conclusion to emphasize the exploratory and hypothesis-generating nature of the study.
- Added clearer wording in the Statistical Analysis section indicating that inferential analyses were exploratory only.
- Reduced the interpretive weight given to chi-square and regression findings in the Results and Discussion.

**5. Reviewer comment**

 In Tables 1 through 6, p-values are repeatedly presented without a clearly defined null hypothesis or clinically meaningful comparative framework. In several instances, the statistical purpose of the chi-square analyses is unclear, particularly because many of the presented variables are descriptive in nature rather than comparative. Additionally, the small expected frequencies in multiple categories may violate assumptions required for chi-square testing. In this context, either Fisher’s exact test or purely descriptive reporting would likely have been more appropriate.

**Response:**

 We thank the reviewer for this important observation and agree that the statistical presentation required further clarification and restraint. In the revised manuscript, descriptive statistics are now clearly presented as the primary analytical framework. We have revised the Statistical Analysis section to state explicitly that chi-square or Fisher’s exact tests were applied only for selected exploratory comparisons between categorical variables, depending on expected cell frequencies and data distribution. We also now define the null hypothesis for these analyses and clarify that any reported p-values are exploratory rather than confirmatory.

 Changes made:

- Clarified that descriptive statistics constitute the primary analytical approach.
- Revised the Statistical Analysis section to specify that chi-square or Fisher’s exact tests were used only where exploratory categorical comparisons were considered clinically relevant.
- Added the statement that the null hypothesis for these exploratory analyses was that no association existed between the variables being compared.
- Added explicit caution that p-values should be interpreted as exploratory only.
- Reduced unnecessary inferential emphasis in tables and text.

**6. Reviewer comment**

 Similarly, the regression analysis should be interpreted with substantial caution. The manuscript does not fully explain how the composite “CAT usage score” was constructed or validated, and the limited sample size raises concerns regarding model stability, overfitting, and reliability of the reported R² values. Although the authors appropriately characterize these findings as exploratory, some statements regarding explanatory power remain somewhat stronger than the available data can robustly support.

**Response:**

 We agree. In the revised manuscript, we clarified that the CAT usage score was derived from questionnaire responses reflecting reported CAT-related experience in orthognathic surgery and that it was created specifically for the purposes of this survey. We now explicitly state that this score is not a previously validated scale. We also further softened the interpretation of regression findings and removed wording that could imply stable explanatory power. The manuscript now clearly states that regression findings are exploratory, vulnerable to instability and overfitting, and should be viewed only as preliminary patterns to inform future studies.

 Changes made:

- Added a clearer explanation of the CAT usage score in the Statistical Analysis section.
- Stated explicitly that the score was survey-derived and not externally validated.
- Added caution regarding limited sample size, sparse data structure, model instability, and overfitting risk.
- Revised Results and Discussion text to avoid overstating explanatory power and to caution interpretation of reported R² values.

**7. Reviewer comment**

 The discussion section is considerably improved; however, it remains somewhat lengthy and occasionally extends beyond the direct findings of the study. Certain sections resemble a narrative literature review rather than a focused interpretation of the collected data. Some speculative statements regarding clinician behavior, digital workflow adoption, and regional sociocultural trends are not directly supported by the study methodology and should therefore be interpreted cautiously.

**Response:**

 We appreciate this important comment and agree. In the revised manuscript, we have further shortened and refocused the Discussion to align more closely with the actual survey findings. Sections that were excessively narrative have been streamlined, and speculative statements regarding clinician behavior, digital workflow adoption, and broader regional trends have been reduced, softened, or removed unless directly supported by the data. The revised Discussion now places greater emphasis on cautious interpretation of the collected findings and their preliminary implications.

 Changes made:

- Reduced the overall length of the Discussion.
- Streamlined literature-heavy sections that were more narrative than interpretive.
- Removed or softened speculative statements not directly supported by the study methodology or findings.
- Refocused the section on interpretation of the observed survey results within an exploratory framework.

**8. Reviewer comment**

 Another conceptual limitation is the broad inclusion of different orthognathic protocols involving CAT, including surgery-first approaches, hybrid protocols, adjunctive TAD usage, and combined fixed appliance mechanics. The questionnaire appears to group these clinical scenarios together without sufficiently distinguishing between them, which may limit the precision of the conclusions.

**Response:**

 We agree and thank the reviewer for highlighting this conceptual limitation. In the revised manuscript, we now explicitly acknowledge that the questionnaire was designed to provide a broad exploratory overview of clinician perspectives and did not distinguish all orthognathic CAT-related protocols with sufficient analytical precision. We also note that future research should evaluate these treatment scenarios separately to allow more protocol-specific interpretation.

 Changes made:

- Added this issue to the questionnaire description in the Methods.
- Added this limitation explicitly to the Limitations section.
- Included a recommendation in the Discussion for future protocol-specific investigations.

**9. Reviewer comment**

**Despite these limitations, the authors should be commended for explicitly acknowledging most methodological weaknesses, including selection bias, low response rate, limited external validity, and the exploratory nature of the statistical analyses. The availability of open data and the transparent reporting of limitations further strengthen the integrity of the work.**

**Response:**

 We sincerely thank the reviewer for this positive assessment. We believe transparency regarding study weaknesses is essential, especially in exploratory work with limited sample size. We have maintained and further clarified these limitations in the revised manuscript and retained the open data statement to support transparency, reproducibility, and future secondary analysis.

**10. Reviewer comment**

**Overall, this manuscript should not be interpreted as providing definitive evidence regarding the use of CAT in orthognathic surgery. However, within the context of an exploratory survey study and considering the publication model of F1000Research, the work provides useful preliminary insights and may serve as a valuable foundation for future multicenter investigations with adequate statistical power.**

**Response:**

 We fully agree. The revised manuscript now consistently presents the study as an exploratory survey offering preliminary insight rather than definitive evidence. We have strengthened this wording in the Abstract, Discussion, Limitations, and Conclusion. We also agree that the current work may serve as an initial foundation for future multicenter studies with stronger methodological design and adequate statistical power.

**11. Reviewer comment**

**Based on the improvements introduced in Version 4 and considering the remaining limitations described above, my recommendation is:**

**Response:**

 We sincerely thank the reviewer for the careful reassessment of Version 4 and for considering the manuscript acceptable in light of the revisions made and the limitations transparently acknowledged.

**12. Reviewer comment**

**Approved with Reservations.**

**Response:**

 We are grateful to the reviewer for this recommendation and for the constructive guidance that helped us improve the manuscript substantially. We have carefully addressed the remaining concerns to the best extent possible within the scope and design of the present study.

**13. Reviewer comment**

**I encourage the authors to consider additional refinement of the statistical presentation and further reduction of speculative discussion sections in future revisions or subsequent related publications.**

**Response:**

 We appreciate this valuable recommendation and have incorporated further refinements in the current revision. Specifically, we improved the statistical presentation by clarifying the exploratory nature of all inferential analyses, defining the null hypothesis for categorical comparisons, specifying the use of chi-square or Fisher’s exact tests as appropriate, and providing clearer caution regarding interpretation of regression findings and R² values. We also further reduced speculative and narrative elements within the Discussion. We agree that these areas will remain important considerations in future related publications.

 We again thank the reviewer for the constructive, balanced, and thoughtful feedback. These comments were highly valuable in strengthening the manuscript, particularly with respect to statistical caution, interpretive restraint, and clearer acknowledgment of conceptual and methodological limitations.

# Reviewer report: Reviewer response for version 3

**DOI:** 10.5256/f1000research.198023.r475308

**Published:** 2026-04-24

**Authors:** Abdelrahman MA Mohamed

**Affiliations:** 1 Orthodontics, Royal College of Surgeons of Edinburgh, Edinburgh, UK

I would like to thank the editor very much for giving me the opportunity to review this paper. Below are my comments and suggestions to strengthen the manuscript.

 The authors did  a great effort to enhance the manuscript, however

1. Title still need to be modified to explain the aim of the study, my suggestion: Awareness and Clinical Experience with Clear Aligners in Orthognathic Surgery: A Saudi Multispecialty Exploratory Survey
2. **Results: **

Author need to define the exact place or better add the link of the Tables s1 and s2 to make it easier for the readers to reach it or add it as normal tables 8 and 9 to the manuscript, also definitions of these table in the footprint need to be added.

**3. Discussion: **The Discussion section is overly long and contains repeated information under different subheadings, leading to unnecessary redundancy.

 I recommend revising it to be more concise and better organized. The Limitations section has a similar issue as discussion.

Is the work clearly and accurately presented and does it cite the current literature?

Partly

If applicable, is the statistical analysis and its interpretation appropriate?

Partly

Are all the source data underlying the results available to ensure full reproducibility?

No

Is the study design appropriate and is the work technically sound?

Partly

Are the conclusions drawn adequately supported by the results?

No

Are sufficient details of methods and analysis provided to allow replication by others?

Partly

Reviewer Expertise:

Orthodontics.

I confirm that I have read this submission and believe that I have an appropriate level of expertise to confirm that it is of an acceptable scientific standard, however I have significant reservations, as outlined above.

# Response

**Published:** 2026-04-25

**Authors:** Ahmad Assari

**Affiliations:** Not Applicable, Saudi Arabia

We sincerely thank the reviewer for their valuable feedback and constructive suggestions to improve the manuscript.

**Comment 1: The title needs modification to better reflect the aim of the study.**

**Response:** Thank you for this suggestion. The title has been revised to better reflect the scope and aim of the study, emphasizing clinician awareness and clinical experience, as well as the exploratory nature of the investigation.

**Comment 2: Clarify the location or accessibility of Supplementary Tables S1 and S2.**

**Response:** We appreciate this important comment. The manuscript has been revised to clearly label “Table S1” and “Table S2” as **Supplementary Tables S1 and S2** throughout the text. A dedicated **Supplementary Materials section** has been added, and references to these tables in the Results section have been clarified to guide readers. Additionally, appropriate explanatory footnotes have been included.

**Comment 3: The Discussion section is overly long and contains repeated information; the Limitations section has similar issues.**

**Response:** Thank you for this constructive observation. The Discussion section has been carefully revised to improve conciseness, reduce redundancy, and enhance overall organization. Repetitive statements have been removed, and the content has been streamlined to focus more directly on interpretation of the study findings in relation to existing literature.

 Similarly, the Limitations section has been substantially revised to eliminate duplication, improve clarity, and present the study constraints in a more concise and structured manner. Redundant phrases related to generalizability and sampling limitations have been removed, and the section has been refined to clearly highlight key methodological limitations, including sample size, exploratory statistical analysis, and potential biases.

 We thank the reviewer again for their insightful comments, which have significantly improved the clarity, structure, and scientific quality of the manuscript.

# Reviewer report: Reviewer response for version 3

**DOI:** 10.5256/f1000research.198023.r475309

**Published:** 2026-04-16

**Authors:** Tiago Fialho

**Affiliations:** 1 Department of Orthodontics, Bauru Dental School, São Paulo University, Bauru, SP, Brazil

I have carefully reviewed the third version (V3) of the manuscript entitled *"The Use of Clear Aligner Therapy for Orthognathic Surgery Patients: A Cross-Sectional Survey Among Saudi Orthodontists and Oral and Maxillofacial Surgeons."* After evaluating the amendments made by the authors in response to previous peer-review rounds, I would like to offer the following assessment regarding its current status and suitability for indexing.

 Firstly, I commend the authors for their diligent efforts in addressing the terminological and structural concerns raised. The correction of the title to "Orthodontists" and the refinement of "dental anomaly" to "dentofacial deformity" in the introduction significantly enhance the academic precision of the text. Furthermore, the inclusion of a detailed flowchart (Figure 1) and the explicit statement of the response rate (16.5%) provide the necessary transparency regarding the study’s recruitment process, which was a point of concern in earlier versions.

 The standardization of tables, including the proper definition of statistical abbreviations and p-values in the footnotes, has also improved the readability and technical quality of the data presentation.

 Regarding the discussion and conclusions, the authors have successfully integrated a more nuanced interpretation of the findings. The clearer distinction between the training needs of orthodontists versus oral surgeons, particularly concerning biomechanics and intermaxillary fixation, adds depth to the study's clinical implications. The conclusion now effectively summarizes the quantitative findings rather than offering generalized statements, which is a vital improvement.

 However, a critical limitation remains: the sample size ($n=46$). Despite the authors' efforts to increase recruitment, the final cohort remains substantially below the initially calculated power requirement ($n=302$). This limitation inherently restricts the generalizability of the findings and the robustness of the inferential statistics. In the F1000Research model, which prioritizes transparency and the publication of all sound scientific work—including exploratory and preliminary data—this does not necessarily preclude publication. The authors have acted appropriately by reframing the study as an "exploratory survey" and being candid about these limitations in the discussion section.

 In conclusion, I believe that the authors have addressed the major technical and editorial queries to the best of their ability within the constraints of their recruitment environment. The manuscript now provides a clear, transparent, and honest account of a preliminary investigation into an emerging clinical field in Saudi Arabia. While the small sample size remains a "reservation," the transparency of the reporting and the open-access availability of the underlying dataset (via Zenodo) align with the journal's standards. Therefore, I recommend that the article be considered for approval, provided that readers continue to interpret the statistical findings with the caution warranted by the exploratory nature of the study.

Is the work clearly and accurately presented and does it cite the current literature?

Partly

If applicable, is the statistical analysis and its interpretation appropriate?

Partly

Are all the source data underlying the results available to ensure full reproducibility?

Yes

Is the study design appropriate and is the work technically sound?

Partly

Are the conclusions drawn adequately supported by the results?

Partly

Are sufficient details of methods and analysis provided to allow replication by others?

Partly

Reviewer Expertise:

Orthodontics; Orthodontic Aligners, Orthodontic mechanics

I confirm that I have read this submission and believe that I have an appropriate level of expertise to confirm that it is of an acceptable scientific standard.

# Response

**Published:** 2026-04-21

**Authors:** Ahmad Assari

**Affiliations:** Not Applicable, Saudi Arabia

We sincerely thank the reviewer for their careful evaluation of Version 3 of our manuscript and for recognizing the substantial improvements made in response to previous review rounds.

 We appreciate the reviewer’s positive feedback regarding the refinement of terminology, improved clarity of the introduction, inclusion of the recruitment flowchart, and enhanced transparency in reporting the response rate and data availability. We are also grateful for the acknowledgment of improvements in table presentation, discussion structure, and the strengthened conclusions.

 Regarding the key concern about the sample size (n = 46 vs. calculated n = 302), we fully agree that this represents a major methodological limitation. In response, we have made the following revisions throughout the manuscript:

- The study has been explicitly repositioned as an exploratory survey in the Abstract, Methods, Results, Discussion, and Conclusion.
- The discrepancy between the calculated and achieved sample size has been clearly stated and consistently emphasized.
- The implications of the small sample size on statistical power, risk of Type I/II errors, and generalizability have been explicitly discussed in the Limitations section.
- All inferential analyses (chi-square and regression) have been clearly described as exploratory, and their interpretation has been appropriately tempered.
- The observed association between age and CAT usage has been reframed as a hypothesis-generating observation rather than a definitive finding.

We have also explicitly clarified that the findings may not be generalizable due to the small sample size and the use of a convenience sampling approach. Additionally, the nature of the dependent variable and the exploratory purpose of the regression model have been clarified to ensure appropriate interpretation. We have also acknowledged potential limitations related to statistical assumptions (e.g., small expected cell counts) and interpreted these analyses with caution.

 In addition, we have strengthened the discussion of sampling limitations, including the use of convenience sampling and the potential for self-selection bias, and we have ensured that all conclusions are aligned with the exploratory nature of the study.

 We appreciate the reviewer’s recognition that the study meets the principles of transparency and open science, particularly through the public availability of the dataset and survey instrument.

 In conclusion, we believe the manuscript now provides a transparent and appropriately cautious account of a preliminary investigation into an emerging clinical topic. We thank the reviewer for their valuable feedback and recommendation for approval with reservations.

# Reviewer report: Reviewer response for version 2

**DOI:** 10.5256/f1000research.191604.r458561

**Published:** 2026-03-10

**Authors:** Abdelrahman MA Mohamed

**Affiliations:** 1 Orthodontics, Royal College of Surgeons of Edinburgh, Edinburgh, UK

I would like to thank the editor very much for giving me the opportunity to review this paper. Below are my comments and suggestions to strengthen the manuscript.

1. Title need to explain the aim of the study
2. **Introduction**:

- The authors need to mention that they are talking abut orthodontic 1 ^st^ and not surgery 1 ^st^ technique, as if it was the surgery 1 ^st^ , there will be no need to revaluate surgeon response.

1. **Material and Methods**:

- Sample size are very small , less than the minimum base on sample size calculation 302 , but the author only included 46 which is much less than the required , especially for the orthognathic surgeons. This needs to be highlighted in the abstract and in conclusion.

1. **Results: **

- I suggest to change the aim to be also the experience of orthodontist in treatment of orthognathic surgery cases also, even with this, the reliability and external validity of the study are compromised.
- WHAT ARE TABLE S1 AND TABLE S2??? IS IT A WRITING MISTAKE OR WHAT???? The error still exists

1. **Discussion:**

- Please Share the reference of this sentence: (Systematic reviews confirm that while CAT is increasingly effective for mild-to-moderate malocclusions, evidence for its use in surgical orthodontics remains limited and largely anecdotal.)?

 - author put facts that do not come from the current study without citing a reference for it, e.g. (Besides, CAT is considered as a popular treatment choice among many orthodontists, however not a common treatment for orthognathic surgery patients.).

 - Discussion writing is not organized, some have large paragraph and some part just have small 1 line sentences. DISCUSSION STILL VERY LONG AND NOT INTERESTING and not majorly focuses on interpreting the results.

 - I see the recommendations have 5 points which can be merged on 2 points.

1. **Conclusion**:

- please make the conclusion shorter.

Is the work clearly and accurately presented and does it cite the current literature?

Partly

If applicable, is the statistical analysis and its interpretation appropriate?

Partly

Are all the source data underlying the results available to ensure full reproducibility?

No

Is the study design appropriate and is the work technically sound?

Partly

Are the conclusions drawn adequately supported by the results?

No

Are sufficient details of methods and analysis provided to allow replication by others?

Partly

Reviewer Expertise:

Orthodontics.

I confirm that I have read this submission and believe that I have an appropriate level of expertise to confirm that it is of an acceptable scientific standard, however I have significant reservations, as outlined above.

# Response

**Published:** 2026-03-19

**Authors:** Ahmad Assari

**Affiliations:** Not Applicable, Saudi Arabia

**Response to Reviewer**

**Comment:** Title need to explain the aim of the study

**Response:**

 Thank you for this valuable suggestion. The title has been revised to more clearly reflect the aim of the study by explicitly including the assessment of knowledge, attitudes, and clinical practices, as well as indicating the exploratory nature of the investigation.

**Comment:**

**Introduction**:

 The authors need to mention that they are talking about orthodontic 1 ^st^ and not surgery 1 ^st^ technique, as if it was the surgery 1 ^st^ , there will be no need to revaluate surgeon response.

**Response:**

 Its use in combination with surgery is emerging; however, its role differs between orthodontics-first and surgery-first protocols. In orthodontics-first approaches, CAT may be used in pre- and post-surgical phases, whereas in surgery-first protocols its role is more limited and evolving.

**Comment:**

**Material and Methods**:

 Sample size are very small , less than the minimum base on sample size calculation 302 , but the author only included 46 which is much less than the required , especially for the orthognathic surgeons. This needs to be highlighted in the abstract and in conclusion.

**Response:**

 Thank you for this important observation. We have revised both the Abstract and Conclusion to explicitly highlight the discrepancy between the calculated minimum sample size (n = 302) and the achieved sample (n = 46). We have also emphasized that this limitation reduces statistical power and affects the robustness of subgroup analyses, particularly for oral and maxillofacial surgeons. The findings are now clearly presented as exploratory and preliminary in nature.

**Comment:**

**Results:**

 I suggest to change the aim to be also the experience of orthodontist in treatment of orthognathic surgery cases also, even with this, the reliability and external validity of the study are compromised.

 WHAT ARE TABLE S1 AND TABLE S2??? IS IT A WRITING MISTAKE OR WHAT???? The error still exists

**Response:**

 Thank you for this valuable feedback. The study aim has been revised in both the Introduction and Abstract to explicitly include **clinical experience** alongside knowledge, attitudes, and practices.

 Thank you for highlighting this issue. We apologize for the lack of clarity. “Table S1” and “Table S2” refer to Supplementary Tables, which contain detailed demographic and clinical data. In the revised manuscript, these have been clearly labeled as “Supplementary Table S1” and “Supplementary Table S2”, and a dedicated Supplementary Material section has been added to improve clarity and accessibility.

 We have also further emphasized the limitations related to sample size and external validity in the revised manuscript to ensure appropriate interpretation of the findings.

**Comment:**

**Discussion:**

 - Please Share the reference of this sentence: (Systematic reviews confirm that while CAT is increasingly effective for mild-to-moderate malocclusions, evidence for its use in surgical orthodontics remains limited and largely anecdotal.)?

 - author put facts that do not come from the current study without citing a reference for it, e.g. (Besides, CAT is considered as a popular treatment choice among many orthodontists, however not a common treatment for orthognathic surgery patients.).

 - Discussion writing is not organized, some have large paragraph and some part just have small 1 line sentences. DISCUSSION STILL VERY LONG AND NOT INTERESTING and not majorly focuses on interpreting the results.

 - I see the recommendations have 5 points which can be merged on 2 points.

**Response:**

 Thank you for these valuable suggestions. We have revised the Discussion section accordingly:

- The previously uncited statement regarding systematic reviews has now been supported with appropriate references.
- Unsupported general statements have been revised or linked directly to the findings of the current study.
- The Discussion has been reorganized to improve flow, reduce redundancy, and place greater emphasis on interpreting the study findings in relation to existing literature.
- Long and fragmented sections have been streamlined for clarity and readability.
- The recommendations section has been condensed from five points into two concise and structured recommendations to improve clarity and impact.

**Comment:**

**Conclusion**:

 - please make the conclusion shorter.

**Response:**

 Thank you for this suggestion. The Conclusion section has been revised to improve conciseness while retaining the key findings and clinical implications. Redundant statements have been removed, and the section now presents a clearer and more focused summary of the study outcomes and future research directions.

# Reviewer report: Reviewer response for version 2

**DOI:** 10.5256/f1000research.191604.r461487

**Published:** 2026-02-25

**Authors:** Tiago Fialho

**Affiliations:** 1 Department of Orthodontics, Bauru Dental School, São Paulo University, Bauru, SP, Brazil

Thank you for the opportunity to review the revised version of this manuscript. The topic is timely and clinically relevant, particularly given the growing incorporation of clear aligner therapy (CAT) into orthognathic surgery protocols. The authors have made appreciable efforts to address prior reviewer comments, especially regarding ethical approval, questionnaire validation, and data transparency. The availability of the dataset and survey instrument is a notable strength and aligns well with the open science principles of the journal.

 Despite these improvements, important methodological and statistical concerns remain that limit the robustness of the conclusions. The manuscript reports a calculated minimum sample size of 302 participants; however, only 46 valid responses were ultimately analyzed. Although the authors attribute this to a limited specialist pool, the manuscript also states that approximately 1,400 eligible professionals exist in the target population. This creates an inconsistency between the theoretical sampling framework and the achieved sample. Most importantly, the final sample size is substantially below the calculated requirement, rendering the study underpowered for inferential statistical testing. Under such conditions, p-values become unstable, and the risk of both Type I and Type II errors increases considerably. If the authors were unable to realistically achieve the required sample size, the study should be explicitly repositioned as an exploratory or pilot investigation, and the inferential claims should be substantially tempered.

 Related to this issue is the use of multiple inferential statistical tests in a small dataset. The manuscript employs several chi-square analyses and univariate linear regression. With a final sample of 46 respondents, there is a high probability that assumptions underlying chi-square tests—particularly minimum expected cell counts—may not be satisfied. Additionally, multiple comparisons are conducted without correction, increasing the risk of false-positive findings. Given the limited sample size, the statistical analysis would be more appropriately framed as primarily descriptive. Inferential modeling, if retained, should be explicitly described as exploratory and interpreted with considerable caution.

 The use of univariate linear regression to assess the association between age and CAT usage also warrants clarification. It is not sufficiently clear whether the dependent variable representing CAT use is continuous, ordinal, or binary. If the outcome is binary or categorical, linear regression is not the appropriate analytical approach, and logistic regression would be methodologically preferable. The reported R² value appears relatively high given the small sample size, which further underscores the need for clarification regarding model specification, variable coding, and assumption testing. Without this clarification, the validity of the regression findings remains uncertain.

 The sampling strategy also limits external validity. The study relied on convenience sampling through online distribution and professional networks, with a response rate of 16.5%. While such an approach is common in survey research, it introduces self-selection bias and may disproportionately attract clinicians already interested in or favorable toward CAT. Although the authors acknowledge this limitation, its implications for generalizability should be more explicitly emphasized in both the discussion and conclusion. Statements regarding trends in CAT adoption should be presented cautiously and framed within the context of these sampling constraints.

 The conclusion that age may influence CAT adoption should be reformulated as a hypothesis-generating observation rather than a definitive finding. Given the limited sample size and statistical fragility, the results should be interpreted as preliminary and in need of confirmation through adequately powered studies. Strengthening the distinction between descriptive observations and inferential claims would improve the internal coherence of the manuscript.

 In summary, the study addresses a relevant and underexplored topic, and its transparency in data reporting is commendable. However, methodological and statistical inconsistencies currently limit the strength of the conclusions. I would recommend approval with reservations contingent upon substantial clarification of the statistical modeling, reconsideration or reframing of the inferential analyses, explicit repositioning of the study as exploratory if appropriate, and a more cautious interpretation of the findings. With these revisions, the manuscript would more accurately reflect the evidentiary weight of the data and would be suitable for publication as an exploratory survey study.

Is the work clearly and accurately presented and does it cite the current literature?

Partly

If applicable, is the statistical analysis and its interpretation appropriate?

Partly

Are all the source data underlying the results available to ensure full reproducibility?

Yes

Is the study design appropriate and is the work technically sound?

Partly

Are the conclusions drawn adequately supported by the results?

Partly

Are sufficient details of methods and analysis provided to allow replication by others?

Partly

Reviewer Expertise:

Orthodontics; Orthodontic Aligners, Orthodontic mechanics

I confirm that I have read this submission and believe that I have an appropriate level of expertise to confirm that it is of an acceptable scientific standard, however I have significant reservations, as outlined above.

# Response

**Published:** 2026-03-22

**Authors:** Ahmad Assari

**Affiliations:** Not Applicable, Saudi Arabia

We sincerely thank the reviewer for their thorough and constructive evaluation of our manuscript. We appreciate the recognition of the study’s relevance, transparency, and data availability. We have carefully addressed all methodological and statistical concerns raised and have revised the manuscript accordingly. Our responses are detailed below.

**1. Sample size and underpowered study**

**Reviewer comment:**

 The manuscript reports a calculated minimum sample size of 302 participants; however, only 46 valid responses were ultimately analyzed. Although the authors attribute this to a limited specialist pool, the manuscript also states that approximately 1,400 eligible professionals exist in the target population. This creates an inconsistency between the theoretical sampling framework and the achieved sample. Most importantly, the final sample size is substantially below the calculated requirement, rendering the study underpowered for inferential statistical testing. Under such conditions, p-values become unstable, and the risk of both Type I and Type II errors increases considerably. If the authors were unable to realistically achieve the required sample size, the study should be explicitly repositioned as an exploratory or pilot investigation, and the inferential claims should be substantially tempered.

**Response:**

 We fully agree with this important observation. The manuscript has been revised to explicitly acknowledge the discrepancy between the calculated and achieved sample size. We have now clearly repositioned the study as an exploratory survey investigation rather than a confirmatory or inferential study.

 This clarification has been added to the Abstract, Methods, Results, Discussion, and Conclusion.

 We have explicitly stated that the limited sample size reduces statistical power and increases the risk of both Type I and Type II errors.

**2. Use of inferential statistical tests in a small dataset**

**Reviewer comment:**

 Related to this issue is the use of multiple inferential statistical tests in a small dataset. The manuscript employs several chi-square analyses and univariate linear regression. With a final sample of 46 respondents, there is a high probability that assumptions underlying chi-square tests—particularly minimum expected cell counts—may not be satisfied. Additionally, multiple comparisons are conducted without correction, increasing the risk of false-positive findings. Given the limited sample size, the statistical analysis would be more appropriately framed as primarily descriptive. Inferential modeling, if retained, should be explicitly described as exploratory and interpreted with considerable caution.

**Response:**

 We agree and have revised the manuscript accordingly:

 All inferential analyses are now explicitly described as exploratory.

 The Results section has been revised to emphasize descriptive findings, with reduced reliance on p-values.

 A clear statement has been added indicating that inferential results should be interpreted as hypothesis-generating rather than confirmatory.

 The limitations of chi-square testing in small samples (including expected cell counts) are now explicitly acknowledged.

**3. Clarification of regression analysis and Sampling strategy and external validity**

**Reviewer comment:**

 The use of univariate linear regression to assess the association between age and CAT usage also warrants clarification. It is not sufficiently clear whether the dependent variable representing CAT use is continuous, ordinal, or binary. If the outcome is binary or categorical, linear regression is not the appropriate analytical approach, and logistic regression would be methodologically preferable. The reported R² value appears relatively high given the small sample size, which further underscores the need for clarification regarding model specification, variable coding, and assumption testing. Without this clarification, the validity of the regression findings remains uncertain. The sampling strategy also limits external validity. The study relied on convenience sampling through online distribution and professional networks, with a response rate of 16.5%. While such an approach is common in survey research, it introduces self-selection bias and may disproportionately attract clinicians already interested in or favorable toward CAT. Although the authors acknowledge this limitation, its implications for generalizability should be more explicitly emphasized in both the discussion and conclusion. Statements regarding trends in CAT adoption should be presented cautiously and framed within the context of these sampling constraints.

**Response:**

 We have clarified the regression methodology in the Statistical Analysis section:

 The dependent variable (CAT usage) is now explicitly described as a continuous composite score derived from questionnaire responses.

 The rationale for using linear regression has been clarified.

 The interpretation of the regression model has been substantially tempered, and results are now presented as exploratory observations rather than definitive findings.

**Convenience sampling and low response rate introduce bias and limit generalizability.**

**Response:**

 We agree and have strengthened this aspect:

 The implications of self-selection bias and non-response bias are now explicitly discussed.

 We have clarified that clinicians with prior interest in CAT may have been overrepresented.

 Statements regarding trends and adoption have been reworded cautiously.

 Limitations in external validity and generalizability are now clearly emphasized in both the Discussion and Conclusion.

**4. Interpretation of age as a predictor**

**Reviewer comment:**

 The conclusion that age may influence CAT adoption should be reformulated as a hypothesis-generating observation rather than a definitive finding. Given the limited sample size and statistical fragility, the results should be interpreted as preliminary and in need of confirmation through adequately powered studies. Strengthening the distinction between descriptive observations and inferential claims would improve the internal coherence of the manuscript.

**Response:**

 This has been revised throughout the manuscript: The association between age and CAT usage is now described as exploratory and hypothesis-generating.

 Any language suggesting a definitive relationship has been removed.

 The need for validation in larger, adequately powered studies has been emphasized.

 We have made the following changes:

 The Results section is now primarily descriptive.

 Inferential findings are clearly labelled as exploratory.

 The Discussion and Conclusion have been revised to ensure cautious interpretation and avoid overgeneralization.

**5. Overall revision**

 In response to the reviewer’s recommendations, the manuscript has been comprehensively revised to:

- Reposition the study as an exploratory survey
- Strengthen transparency regarding methodological limitations
- Improve clarity of statistical methods
- Ensure appropriate interpretation of findings

We are grateful for the reviewer’s insightful feedback, which has significantly improved the methodological clarity and scientific rigor of the manuscript. We believe the revised version now more accurately reflects the exploratory nature of the study and provides a balanced and cautious interpretation of the findings.

# Reviewer report: Reviewer response for version 1

**DOI:** 10.5256/f1000research.187514.r419152

**Published:** 2025-10-22

**Authors:** Abdelrahman MA Mohamed

**Affiliations:** 1 Orthodontics, Royal College of Surgeons of Edinburgh, Edinburgh, UK

I would like to thank the editor very much for giving me the opportunity to review this paper. Below are my comments and suggestions to strengthen the manuscript.

1. **Title  :**

It should be *orthodontists,* not *orthodontics*.

1. Abstract:

The response rate percentage (16.5%) should appear in the results.

1. **Introduction**:

- The authors in the 1 ^st^ line mentioned ‘ dental anomaly’ it would be better to change it with ‘dentofacial’ as they are talking about skeletal deformities and orthognathic surgery.
- focus more on the gap in literature concerning CAT in orthognathic surgery.

1. **Material and Methods**:

- Better to put the ethical consideration at the beginning of the methods section and merge it with the study design and setting.
- Ethical approval is mentioned twice ( in methods and before the references with slight variation in details) better to be accurately mentioned once.
- How participants were recruited? (email list? Saudi Orthodontic society? Or how?).
- Can the author put the equation used in the Sample size calculation?
- Sample size are very small especially for the orthognathic surgeons.
- Author did not mention how to handle the bias came from the very low response rate (16.5%).
- The authors mentioned that the sample size required is 278, but they only included 46, this is too small sample size compared to the actual needed sample based on the authors calculation. This can extremely weaken the study by:
  - Reduced statistical power (less ability to detect real effects).
  - Increased risk of Type II error (failing to detect a difference that exists).

- Many orthodontist don’t treat the cases with orthodontic-orthognathic surgery, how about even treated these cases with CAT technology, this required a good experience in orthodontic-orthognathic surgery cases. It would be better to chooses expert orthodontist and maxillo-facial surgeons in this field in the inclusion exclusion criteria.

 - In the statistical step, authors mentioned  that 4 participants did not complete their response, can the author give the actual number of participants whom completed the response and included in the study?

 - Why did the  authors used Mann-Whitney U test and not independent samples t-test? Did they performed the normal distribution analysis and found that the data are non-parametric ( not normally distributed) please explain.

 - It is better to explain the program used in the statistics SPSS in the beginning and not the end of the statistical analysis.

 - In the statistical analysis: author should mention the type of statistical test used above each table and cite each statistical test to the corresponding table.

1. **Results: **

- Ensure all abbreviations are defined in table footnotes ( p-value, CI, etc).
- What are Table S1 and Table S2??? Is it a writing mistake or what?
- Many sentences in the 1 ^st^ paragraph are not cited and linked to any tables, better to arrange it in a table.

1. **Discussion:**

- At the end of the discussion, the author mention that: ‘ *Orthodontists and oral & maxillofacial surgeons should have catered CATs in their postgraduate training. They should expand on digital workflows, aligner biomechanics, and technology integration, and allocate time to the importance of interdisciplinary cooperation, clinical sequencing, and the utility of hybrid approaches when CAT alone may prove inadequate’ *I think this paragraph is not accurate, this is correct for orthodontist but not for orthognathic surgeons to know the biomechanics at all, clinical sequence nor utility of hybrid approach, it is much simple for orthognathic surgeon in this field just to know basks and to know hot to apply the inter-maxillary elastics after surgery  for patient wearing CAT.

 - Beside the discussion looks long, it give a little explanation of the founded result. I recommend the author to focus more on the discussion section in explanation of the finding of the results and compare it more with other studies and findings in the literature.

 - Put practical recommendations (e.g., training courses, inclusion of CAT in residency programs).

1. **Conclusion** :

- Generally,the conclusions, seems giving no real benefit, the author should also mention the difficulties during orthognathic surgery when used with CAT, e.g. the maxillary fixation, how to use the heavy intermaxillary elastics, splints etc., not only mentioning the orthodontic difficulties.

 - Should summarize major quantitative findings and their implications e.g.: “Most orthodontists and surgeons surveyed had limited experience with CAT in orthognathic cases, indicating the need for enhanced clinical training and interdisciplinary collaboration.”

1. **References** :

- The introduction started with an odd statement that not linked to the study , and in return the selected reference are not linked to the study at all ???!!!  ref no. 1-3.

 - Where did the author cite reference no. 43? Also this reference almost have same idea , patient of the current study?

1. **Tables:**

- Table 1 is unclear and it’s column 2 is not defined.

 - Many variables ( rows) in table 2 seems not linked to the CAT with surgery, it is only related to CAT and orthodontics. e.g.: row 3: *It is challenging to achieve the needed pre surgical space closure, row 4 *

1. Minor grammar and style edits are recommended.

Is the work clearly and accurately presented and does it cite the current literature?

Partly

If applicable, is the statistical analysis and its interpretation appropriate?

Partly

Are all the source data underlying the results available to ensure full reproducibility?

No

Is the study design appropriate and is the work technically sound?

Partly

Are the conclusions drawn adequately supported by the results?

No

Are sufficient details of methods and analysis provided to allow replication by others?

Partly

Reviewer Expertise:

Orthodontics.

I confirm that I have read this submission and believe that I have an appropriate level of expertise to confirm that it is of an acceptable scientific standard, however I have significant reservations, as outlined above.

# Response

**Published:** 2025-11-16

**Authors:** Ahmad Assari

**Affiliations:** Not Applicable, Saudi Arabia

1. **Title  :**

It should be *orthodontists,* not *orthodontics*.

**Response:** done

1. **Abstract **:

The response rate percentage (16.5%) should appear in the results.

**Response:** done

  

1. **Introduction**:

- The authors in the 1 ^st^ line mentioned ‘dental anomaly’ it would be better to change it with ‘dentofacial’ as they are talking about skeletal deformities and orthognathic surgery.

**Response:**

 We thank the reviewer for this helpful suggestion. The term **“dental anomaly”** has been replaced with **“dentofacial anomaly”** to more accurately reflect the skeletal and facial components relevant to orthognathic surgery. This modification ensures better alignment between the introductory context and the study’s clinical focus.

- focus more on the gap in literature concerning CAT in orthognathic surgery.

Response: done

**Response:**

 We agree with the reviewer’s observation and have revised the final paragraph of the Introduction to better highlight the **literature gap** on the use of Clear Aligner Therapy (CAT) in orthognathic surgery. The new text emphasizes that, although CAT has gained global popularity in orthodontics, there is a **lack of empirical data** regarding its application, feasibility, and clinician readiness in surgical orthodontic contexts, especially within the Saudi population. This strengthened focus now clearly establishes the rationale for conducting the present study.

1. **Material and Methods**:

- Better to put the ethical consideration at the beginning of the methods section and merge it with the study design and setting.

**Response:**

 We appreciate the reviewer’s recommendation. The **Ethical Consideration** subsection has now been **moved and merged** with the **Study Design and Setting** subsection at the beginning of the Methods section to improve logical flow and eliminate redundancy.

- Ethical approval is mentioned twice ( in methods and before the references with slight variation in details) better to be accurately mentioned once.

**Response:**

 We thank the reviewer for identifying this duplication. The repeated ethical statement before the References section has been **removed**, and only the version integrated within the **Study Design and Setting** subsection has been retained for accuracy and consistency.

- How were participants recruited? (email list? Saudi Orthodontic society? Or how?).

**Response:**

 Thank you for this important clarification. The **participant recruitment process** has now been described in detail. The revised text specifies that survey invitations were distributed through **professional email lists**, **the Saudi Orthodontic Society**, **academic institutions**, and **social media platforms** (e.g., WhatsApp and LinkedIn) to maximize geographic and professional reach.

- Can the author put the equation used in the Sample size calculation?

**Response:**

 We appreciate the suggestion. The **finite population sample size equation** has now been added to the *Sample Size and Population* subsection, including variable definitions for clarity.

- Sample size are very small especially for the orthognathic surgeons.

**Response:**

 We agree with the reviewer. A statement has been added acknowledging the **small subgroup size** of oral and maxillofacial surgeons, attributing it to their limited national representation and demanding clinical schedules. The revision also clarifies that while the sample size limits inferential generalization, it provides **valuable pilot data** for future multicenter and higher-powered studies.

- Author did not mention how to handle the bias came from the very low response rate (16.5%).

**Response:**

 We thank the reviewer for noting this. A paragraph addressing **potential non-response and self-selection bias** has been added to the *Sample Size and Population* subsection. The revised text explains measures taken to minimize bias (multi-channel recruitment, anonymity assurance, and non-parametric analysis) and justifies interpreting results within an **exploratory framework** rather than population-level inference.

- The authors mentioned that the sample size required is 278, but they only included 46, this is too small sample size compared to the actual needed sample based on the authors calculation. This can extremely weaken the study by:
  - Reduced statistical power (less ability to detect real effects).
  - Increased risk of Type II error (failing to detect a difference that exists).

**Response:**

 We acknowledge this limitation and have now included an explicit statement describing the **impact of reduced sample size on statistical power and Type II error**. This limitation is now clearly discussed in the *Sample Size and Population* subsection and reiterated in the *Limitations* section, clarifying that this study was **exploratory** and intended to provide **pilot-level evidence** to guide larger-scale future research.

 - Many orthodontist don’t treat the cases with orthodontic-orthognathic surgery, how about even treated these cases with CAT technology, this required a good experience in orthodontic-orthognathic surgery cases. It would be better to chooses expert orthodontist and maxillo-facial surgeons in this field in the inclusion exclusion criteria.

**Response:**

 We thank the reviewer for this insightful observation. We have now **clarified the inclusion criteria** to emphasize that the study targeted **licensed orthodontists and oral and maxillofacial surgeons actively practicing in Saudi Arabia**, regardless of their level of experience with orthognathic surgery.

 However, to address the reviewer’s concern, we have added a statement specifying that **future studies should consider recruiting only experienced clinicians or specialists who regularly perform orthognathic treatments**, to ensure deeper insight into expert practices. This note has been added to the *Limitations* section to acknowledge the potential variability in participant expertise.

 - In the statistical step, authors mentioned  that 4 participants did not complete their response, can the author give the actual number of participants whom completed the response and included in the study?

**Response:**

 We appreciate this request for clarity. The total number of participants who **completed the questionnaire in full and were included in the final analysis was 46**, after excluding **4 incomplete responses** from the original 50 submissions. This has been explicitly stated in the *Statistical Analysis* subsection.

 - Why did the  authors used Mann-Whitney U test and not independent samples t-test? Did they performed the normal distribution analysis and found that the data are non-parametric ( not normally distributed) please explain.

**Response:**

 We thank the reviewer for highlighting this point. The **Mann–Whitney U test** was used because the **normality assumption was not met** for the knowledge score data. This indicates that the data were **non-normally distributed**, making the Mann–Whitney U test a more appropriate non-parametric alternative to the independent samples t-test.

 This clarification has been added to the *Statistical Analysis* section to ensure transparency regarding the choice of statistical test.

 - It is better to explain the program used in the statistics SPSS in the beginning and not the end of the statistical analysis.

**Response:**

 We agree with the reviewer. The mention of the statistical software ( **IBM SPSS Statistics version 27, Chicago, IL, USA**) has been **relocated to the beginning** of the *Statistical Analysis* section for improved readability and consistency with journal standards.

 - In the statistical analysis: author should mention the type of statistical test used above each table and cite each statistical test to the corresponding table.

**Response:**

 We appreciate this helpful suggestion. The **type of statistical test** used in each analysis has now been **explicitly indicated above every table** in the Results section (e.g., *Statistical test: Chi-square test*, *Statistical test: Mann–Whitney U test*, *Statistical test: Univariate linear regression analysis*).

 Additionally, cross-references have been added in the *Statistical Analysis* section to identify which test corresponds to which table for greater clarity and transparency.

1. **Results:**

- Ensure all abbreviations are defined in table footnotes ( p-value, CI, etc).

**Response:**

 We appreciate the reviewer’s careful attention to detail. All abbreviations, including *p-value* (probability value) and *CI* (confidence interval), have now been **clearly defined in the footnotes of all tables (Tables 1–7)** to ensure clarity and uniformity.

- What are Table S1 and Table S2??? Is it a writing mistake or what?

**Response:**

 We thank the reviewer for pointing out this issue. The mention of **“Table S1” and “Table S2”** was an **inadvertent labeling error** carried over from an earlier draft where supplementary tables were planned but later merged into the main text. These have now been **corrected and relabeled** as **Table 1 and Table 2**, respectively, ensuring consistency throughout the Results section and in-text citations.

 All references to “S1” and “S2” have been deleted and replaced with “Table 1” and “Table 2.”

- Many sentences in the 1 ^st^ paragraph are not cited and linked to any tables, better to arrange it in a table.

**Response:**

 We appreciate this constructive comment. The first paragraph of the Results section has been **restructured for clarity**. Quantitative findings that were previously written narratively are now **directly linked to their corresponding tables**, and where appropriate, data have been reorganized into concise tabular form.

 Each descriptive statement now includes the relevant table reference in parentheses, e.g., “(Table 1)” or “(Table 2),” to allow readers to easily cross-check findings.

 This restructuring improves logical flow, clarity, and data traceability.

1. **Discussion:**

- At the end of the discussion, the author mention that: ‘ *Orthodontists and oral & maxillofacial surgeons should have catered CATs in their postgraduate training. They should expand on digital workflows, aligner biomechanics, and technology integration, and allocate time to the importance of interdisciplinary cooperation, clinical sequencing, and the utility of hybrid approaches when CAT alone may prove inadequate’ *I think this paragraph is not accurate, this is correct for orthodontist but not for orthognathic surgeons to know the biomechanics at all, clinical sequence nor utility of hybrid approach, it is much simple for orthognathic surgeon in this field just to know basks and to know hot to apply the inter-maxillary elastics after surgery  for patient wearing CAT.

**Response:**

 We thank the reviewer for this precise and valuable clarification. We agree that the original statement overstated the training requirements for oral and maxillofacial surgeons. The paragraph has been **revised to clearly distinguish between the educational needs of orthodontists and surgeons.**

 Orthodontists’ training recommendations remain focused on **digital workflows, biomechanics, and case sequencing**, while the surgeons’ component now emphasizes **understanding splint adaptation, intermaxillary fixation (IMF), and postoperative elastic management**.

 - Beside the discussion looks long, it give a little explanation of the founded result. I recommend the author to focus more on the discussion section in explanation of the finding of the results and compare it more with other studies and findings in the literature.

**Response:**

 We appreciate this insightful feedback. The **Discussion section has been condensed and refocused** to emphasize interpretation of our main results rather than general literature review.

 - Put practical recommendations (e.g., training courses, inclusion of CAT in residency programs).

**Response:**

 Thank you for this excellent suggestion. We have incorporated a new **“Practical Recommendations” paragraph** at the end of the Discussion, explicitly outlining **actionable educational and clinical strategies.** These include integrating CAT education into orthodontic and surgical residency curricula, conducting continuing professional training workshops, and fostering national and international collaboration.

1. **Conclusion** :

- Generally,the conclusions, seems giving no real benefit, the author should also mention the difficulties during orthognathic surgery when used with CAT, e.g. the maxillary fixation, how to use the heavy intermaxillary elastics, splints etc., not only mentioning the orthodontic difficulties.

**Response:**

 We thank the reviewer for this valuable feedback. We have revised the **Conclusion** to provide a more comprehensive and clinically relevant summary that acknowledges both **orthodontic and surgical challenges** associated with Clear Aligner Therapy (CAT) in orthognathic surgery.

 The revised version now includes mention of **intraoperative and postoperative challenges**, such as **maxillary fixation, splint adaptation, and management of intermaxillary elastics** in CAT-treated patients, to ensure that both specialties’ perspectives are reflected.

 - Should summarize major quantitative findings and their implications e.g.: “Most orthodontists and surgeons surveyed had limited experience with CAT in orthognathic cases, indicating the need for enhanced clinical training and interdisciplinary collaboration.”

**Response:**

 We fully agree with this suggestion. The revised conclusion now incorporates **quantitative summary statements** (e.g., the low response rate, limited experience levels, and lack of significant knowledge difference between specialties) and their broader **implications for clinical practice and training**.

 The final paragraph also emphasizes the importance of **interdisciplinary collaboration**, **standardized training**, and **evidence-based clinical integration of CAT** into surgical orthodontics.

1. **References** :

- The introduction started with an odd statement that not linked to the study , and in return the selected reference are not linked to the study at all ???!!!  ref no. 1-3.

**Response:**

 We thank the reviewer for this observation. We respectfully clarify that **References 1–3** were intentionally included to provide epidemiological context regarding the **prevalence of dentofacial anomalies**, which are the underlying conditions often treated through **orthognathic surgery**—the core topic of this study. However, to improve clarity and ensure stronger linkage between these references and the study focus, the **opening paragraph of the Introduction** was **revised **to better connect the cited data on dentofacial anomalies to the relevance of orthognathic surgery and, subsequently, to Clear Aligner Therapy (CAT).

 - Where did the author cite reference no. 43? Also this reference almost have same idea , patient of the current study?

**Response:**

 We thank the reviewer for this valuable observation. Reference no. 43 refers to the **Zenodo data repository** containing the dataset that supports the findings of the current study, rather than to a separate research article or independent sample.

1. **Tables:**

- Table 1 is unclear and it’s column 2 is not defined.

**Response:**

 We thank the reviewer for identifying this oversight. The **second column in Table 1** has now been properly labeled to specify its content.

 - Many variables ( rows) in table 2 seems not linked to the CAT with surgery, it is only related to CAT and orthodontics. e.g.: row 3: *It is challenging to achieve the needed pre surgical space closure, row 4*

***Response:***

*We appreciate the reviewer’s observation. We agree that certain items in **Table 2** appeared more related to **general orthodontic CAT practice** rather than **surgical applications**.*
